# GATOR1 complex controls cisplatin sensitivity

**DOI:** 10.1038/s41419-025-08392-4

**Published:** 2025-12-30

**Authors:** Zhenrui Pan, Hanxiao Zhang, Xia Xiao, Catherine Brenner, Svetlana Dokudovskaya

**Affiliations:** 1 CNRS UMR9018, Université Paris-Saclay, Gustave Roussy, Villejuif, France; 2 Present Address: Department of Pathophysiology, School of Basic Medical Sciences, College of Medicine, Zhengzhou University, Zhengzhou, Henan China

**Keywords:** Tumour-suppressor proteins, Stress signalling

## Abstract

Cisplatin administration is the primary chemotherapy approach for many epithelial cancers. However, resistance to this drug poses a significant challenge to effective treatment. Despite the identification of numerous factors associated with resistance, reliable biomarkers predicting drug response remain elusive. Previously, low expression of the NPRL2 tumor suppressor was linked to cisplatin resistance. NPRL2, along with NPRL3 and DEPDC5, forms the GATOR1 complex, an upstream regulator of the mTORС1, the function of which is perturbed in many cancers, particularly those resistant to cisplatin. Here, we compare non-cancerous bronchial epithelium BEAS-2B cells with GATOR1 deletions, serving as a model of intrinsic cisplatin resistance, with non-small cell lung cancer lines A549, H460, and H1975 with acquired resistance to the drug. We found that deletion of any GATOR1 member, not solely NPRL2, promotes cisplatin resistance, whereas their overexpression renders cells sensitive to the drug. In cells with GATOR1 deletions, expression of the ATP7A transporter required for cisplatin efflux is increased, while expression of cisplatin influx transporters CTR2 and LRRC8A is downregulated, especially after treatment with the drug. This hinders drug accumulation in cells, resulting in the formation of fewer cisplatin-DNA adducts. Simultaneously, these cells exhibit enhanced DNA damage response and mTORC1 activity. Overexpression of GATOR1 components and/or concomitant treatment with an mTORC1 inhibitor restores sensitivity to cisplatin. Transcriptomic analysis of GATOR1-deleted BEAS-2B cells, treated or not with the drug, identifies new signatures important for understanding GATOR1 function and its role in cisplatin resistance. Thus, GATOR1 not only participates in the cellular response to amino acid availability but also plays a role in resistance to DNA-damaging anticancer drugs. This novel function of GATOR1 should be taken into account when developing new strategies to combat chemoresistance.

## Introduction

Cisplatin was approved by the FDA as an anticancer agent in 1978 and has since remained one of the most widely used drugs for treating various carcinomas, including those derived from the bladder, testes, cervix, breast, and ovaries [1, 2]. Additionally, cisplatin is the standard first-line treatment for lung cancer, the leading cause of cancer-related deaths (17%) globally, the most prevalent cancer type in men, and the third most common in women [3]. Lung cancer is broadly classified into two major groups: small cell lung cancer (SCLC) and non-small cell lung cancer (NSCLC), with NSCLC accounting for approximately 80–85% of all cases. NSCLC patients generally exhibit lower sensitivity to cisplatin compared to those with SCLC [4].

Cisplatin and its derivatives enter cells by passive diffusion and by active transport through volume-regulated anion channels [5] and copper influx transporters [6]. In the cytoplasm, cisplatin becomes aquated due to lower chloride concentrations compared to the bloodstream. Subsequently, this highly electrophilic molecule interacts with nucleic acids, proteins, and lipids [7].

The primary targets of cisplatin are nuclear and mitochondrial DNA, where the drug forms intra- and interstrand crosslinks with purine bases. If such DNA damage is not resolved by DNA repair pathways, it impedes DNA replication, consequently affecting mRNA and protein synthesis. Moreover, cisplatin triggers the production of reactive oxygen species (ROS), leading to lysosomal destruction and the release of lysosomal proteases. Cisplatin also induces endoplasmic reticulum (ER) stress, causing calcium storage dysregulation and the formation of misfolded proteins [8]. All these disturbances activate several signaling pathways, ultimately culminating in necrosis or apoptosis [9].

Although cisplatin is used as a first-line treatment in many cancers, its application is hampered by severe side effects, along with intrinsic and acquired resistance. There are still no effective therapies for the further treatment of patients who develop resistance. Multiple factors have been found to lead to resistance to cisplatin. The most important are: reduced drug accumulation due to either decreased influx or increased efflux (or both); inactivation of the drug by binding with different proteins (e.g., with glutathione); increased DNA repair; changes in DNA damage response; alteration of apoptosis signaling pathways [10]. Recent findings also connect cisplatin resistance with metabolic signaling networks, especially autophagy and the mTORC1 pathway [11].

The highly conserved mammalian target of rapamycin (mTOR) is a serine/threonine protein kinase belonging to the PI3K-related kinase (PIKK) family. As a part of mTOR Complex 1 (mTORC1), mTOR regulates cellular responses to many stresses and maintains cellular homeostasis. To ensure optimal growth and metabolism, mTORC1 integrates signals from many intracellular and extracellular cues, including amino acids, growth factors, energy, and DNA damage, relying on numerous upstream regulators and downstream effectors [12, 13]. In healthy cells, active mTORC1 inhibits autophagy, while cisplatin treatment inhibits mTORC1 activity and induces autophagy. Cancer cells resistant to cisplatin generally exhibit elevated mTORC1 activity, but autophagy can be either enhanced or suppressed, depending on the cancer type [11, 14, 15].

One of the major upstream regulators of the mTORC1 pathway, the GATOR1 complex, is involved in nutrient sensing and responding [16]. Composed of three proteins (NPRL2, NPRL3, and DEPDC5), GATOR1 inhibits mTORC1 by acting as a GTPase-activating protein (GAP) for the mTORC1 regulator RAG GTPase A [16–19]. Various mutations in the genes encoding GATOR1 components have been identified in many cancers and epilepsies [17]. Low expression of NPRL2 in NSCLC is correlated with resistance to cisplatin [20]. Overexpression of NPRL2 in NPRL2-deficient and cisplatin-resistant NSCLC cells reactivated the cellular response to cisplatin and promoted tumor suppression activity in vitro and in mouse models [20], which might be linked to the role of overexpressed NPRL2 in promoting DNA damage and ROS production [21]. However, the precise molecular mechanisms underlying NPRL2’s role in cisplatin resistance remain unclear. While NPRL3 (a paralogue of NPRL2) and DEPDC5 are members of the same complex, there have been no reports thus far on whether the expression of these proteins affects cisplatin sensitivity.

Here, we demonstrate that deletion of any member of the GATOR1 complex confers resistance to cisplatin by upregulating the efflux transporter ATP7A, downregulating the influx transporters CTR2 and LRRC8A, altering DNA damage response, and affecting mTORC1 pathway function. We also compare non-cancerous cells with GATOR1 deletions, serving as a model of intrinsic cisplatin resistance, with several NSCLC lines with acquired resistance to the drug, analyzing changes in the transcriptome and functioning of signaling pathways. Our findings reveal that despite being part of the same complex, each GATOR1 member exhibits distinct functional characteristics in regulating the transcriptome, mTORC1 pathway, and DNA damage response.

## Materials and methods

### Reagents, chemicals and kits

Dulbecco’s Modified Eagle Medium, DMEM (31966-021, Gibco), was used for cultivating the cell lines used in this study. NuPAGE 4–12% Bis–Tris gels (NP0322), 20× MOPS SDS running buffer (NP0001), LDS sample buffer (NP0007), RevertAidTM H Minus Reverse Transcriptase (EP0451), Maxima™ H Minus cDNA Synthesis Master Mix (15676019), Powerup SYBR Green Master Mix (A25742), SYBR Gold staining solution (S11494), and MTT (M6494) were from Thermo Fisher Scientific. 70% HNO_3_ (225711) and MNase (N3755) were from Sigma. The phosphatase inhibitor cocktail (04 906 837 001) was from Roche. The Immobilon-P PVDF membrane (IPVH00010) and ECL reagent (WBKLS0500) were from Merck/Millipore. Cisplatin was produced by Mylan (3400956318575). Torin 1 (HY-13003) was from MedChemExpress. Proteinase K (750506), the NucleoSpin tissue kit (740952), and the NucleoSpin RNA kit (740955) were from Macherey-Nagel. The FITC Annexin V apoptosis detection kit with 7-AAD (TNB35-6410-KIT) was from TONBO Bioscience.

### Antibodies

The antibodies were obtained from the following sources: anti-NPRL3 (HPA011741), anti-FLAG (F3165), anti-β-actin (A1978), and anti-rat IgG-HRP (A9037) were from Sigma; anti-GFP (11814460001) was from Roche; anti-ATP7A (D9) (SC-376467) and anti-ATP7B (A11) (SC373964) were from Santa-Cruz. Anti-NPRL2 (37344), anti-CTR1 (13086), anti-phospho-p70 S6 kinase (Thr389) (9206), anti-p70 S6 kinase (9202), anti-p4EBP1 (Thr37/46) (2855), anti-4EBP1 (9452), anti-pTFEB (Ser211) (37681), anti-TFEB (37785), anti-pULK1 (Ser757) (14202), anti-ULK1 (8054), anti-pS6 (Ser 235/236) (2211), anti-S6 (2317), anti-p62 (8025), anti-phospho-histone-H2AX Ser139/Tyr142 (5438), anti-PARP1 (9542), anti-pAKT Ser473 (4060), anti-AKT (4619), anti-pAMPKα (Thr172) (2535), anti-AMPKα (5831), anti-phospho-p53 (Ser15) (9284), anti-p53 (2524), anti-pATM S1981 (4526), anti-ATM (2873), anti-ATR (2790), anti-pCHK1 (S345) (2341), anti-CHK1 (2360), anti-pCHK2 (Thr68) (2661), anti-CHK2 (2662), anti-p21 (2946), anti-LC3B (2775), anti-caspase 3 (9662), anti-GAPDH (2118), anti-vinculin (18799) were from Cell Signaling Technology. Anti-PAR antibody (ALX-804-220-R100) was from Enzo Life Sciences. Anti-CTR2 (PA5-22961) was from Life Technologies/Thermo Fisher Scientific. Anti-cisplatin-modified DNA antibody [CP9/19] (ab103261), anti-pATR (Thr1989) (ab227851), anti-LRRC8A (ab254389), anti-ATP7A (ab308524), anti-DEPDC5 (ab213181), and anti-p73 (ab215038) antibodies were from Abcam. HRP-labeled anti-mouse (315035003) and HRP-labeled anti-rabbit (111035144) antibodies were from Jackson ImmunoResearch.

### Plasmids

The plasmid pc5FLAG-wt-humanTUSC4 (pFLAG-NPRL2) and empty vector pc5FLAG were from Dr. Fujita (Cancer Chemotherapy Center of JFCK, Tokyo, Japan). pFLAG-DEPDC5 was a generous gift from Stephanie Baulac, ICM, Paris. pFLAG-NPRL3 plasmid was obtained by subcloning of the NPRL3 gene from pcDNA3-HA-C16orf35 [21, 22] to pc5FLAG on EcoRI and XhoI sites, using the following primers:

NPRL3-5-N-term FLAG (EcoRI):

CGATCGGAATTCTGCTGATGGAAATGAAGGTCCTCAGTCCCCATTC

NPRL3-3-N-term FLAG (XhoI):

CCATCGCTCGAGTCAGGGGAGCAGAGCCTGGAAGACGGCAATGAC.

### Cell lines and cell culture

BEAS-2B cells were obtained from ATCC. Cisplatin-sensitive A549 cells and cisplatin-resistant A549 cells were obtained through an MTA agreement with the University of Kent, UK [23]. Cisplatin-sensitive and resistant H460 cells, and cisplatin-sensitive and resistant H1975 cells [15] were kindly provided by Dr. Michael Wanzel (Institute of Molecular Oncology, Philipps-University of Marburg, Germany). BEAS-2B, A549, and H460 were derived from male patients, and H1975 was derived from a female patient. Cells were cultured in DMEM supplemented with 10% fetal bovine serum, 1% penicillin–streptomycin, and maintained in a humidified incubator at 37 °C and 5% CO_2_. Cells were regularly tested for the presence of mycoplasma.

### Generation of BEAS-2B cell lines with deletions of GATOR1 components

To generate GATOR1-deleted BEAS-2B cell lines, a pair of single guide RNAs targeting NPRL2 or NPRL3, or DEPDC5 was designed and cloned into the phU6-gRNA (Addgene, 53188). BEAS-2B cells were co-transfected with phU6-NPRL2 gRNA, phU6-NPRL3 gRNA, or phU6-DEPDC5 gRNA and CRISPR-Cas9-EGFP. After 48 h, GFP-positive cells were sorted in 96-well plates by flow cytometry using an ARIA Fusion-UV (BD Bioscience). Cells were allowed to grow for up to two weeks, and clones without GATOR1 expression were selected by western blot analysis.

Primers for sgRNAs:

NPRL2-sgRNA1-Forward: TTCTTGTGTTCGATGCACAC

NPRL2-sgRNA1-Reverse: GTGTGCATCGAACACAAGAA

NPRL3-sgRNA1-Forward: TGTTGTCCCGCATCCCGCCG

NPRL3-sgRNA1-Reverse: CGGCGGGATGCGGGACAACA

DEPDC5-sgRNA-Forward: GGAACACTTGAGTCACAGTC

DEPDC5-sgRNA-Reverse: GACTGTGACTCAAGTGTTCC

### MTT assay

10,000 cells per well were seeded in a 96-well plate and treated with cisplatin for 24 h. Medium was replaced with 100 μl of fresh culture medium, 20 μl of the 12 mM MTT solution was added to each well, and the plate was incubated for 4 h at 37 °C. After incubation, 100 μl of 10% SDS–0.01 N HCl solution was added to each well to dissolve the produced formazan and incubated overnight at 37 °C in a humidified chamber. The absorbance of the converted dye was measured at 570 nm using an ELISA plate reader. IC_50_ was calculated using GraphPad Prism software.

### Apoptosis analysis

0.3 × 10^6^ cells were seeded in a six-well plate, and after 18–24 h of growth, cells were treated with 50 μΜ cisplatin for 24 h. Cells were collected by trypsinization, centrifuged at 400×*g* for 5 min at room temperature, and the supernatant was discarded. The pellet was resuspended in 1 ml of stain buffer from FITC Annexin V Staining Kit, centrifuged at 400×*g* for 5 min at room temperature, and the supernatant was discarded. Pellet was resuspended to a concentration of 1 × 10^6^ cells/ml in Annexin V Binding Buffer, and 100 μl of cell suspension was placed in an individual tube for staining. 5 μl of FITC Annexin V and 5 μl of 7-AAD solution were added to a sample, mixed gently, incubated at room temperature in the dark for 15 min, and 400 μl of Annexin V Binding Buffer was added to each tube. Samples were analyzed by flow cytometry.

### Colony formation assay

A total of 3000 cells per well were seeded into six-well plates and maintained in growth medium for 7 days. Wells assigned to the treatment group were then replaced with medium containing 10 µM of cisplatin, while control wells received fresh medium. After 4 days, the medium was refreshed, and the same conditions were reapplied (10 µM cisplatin for treated wells; fresh medium for controls) for an additional 3 days. Following treatment, cultures were washed twice with 1× PBS, fixed for 30 min with 4% paraformaldehyde (1 ml per well), rinsed twice with 1× PBS, and stained for 15 min with crystal violet (250 mg/100 ml in 90% methanol, 10% formaldehyde). Excess dye was removed by rinsing with water, and plates were allowed to air-dry completely. Images of stained colonies were acquired using an Amersham ImageQuant 800 imaging system (Cytiva) in colorimetric/OD mode. Colony quantification was performed using ImageJ, with identical threshold settings applied across all images within a group. For wells in which maximum colony coverage was <30% of the well area, individual colonies were counted. When coverage exceeded 30%, total colony area was measured instead.

### Transfection

BEAS-2B cells were seeded at 1 × 10^5^ cells per well in six-well plates and cultured for 24 h prior to transfection. Cells were transfected with plasmids encoding Flag-NPRL2, Flag-NPRL3, or Flag-DEPDC5 using ViaFect™ Transfection Reagent (Promega, E4981) according to the manufacturer’s instructions. For each well, 100 ng plasmid DNA was mixed with ViaFect at a 1:3 (µg:µl) DNA-to-reagent ratio in Opti-MEM™ I Reduced Serum Medium (Gibco, 31985-062) to a final volume of 60 µL, incubated for 20 min at room temperature, and added dropwise to cells in 1.8 mL DMEM. After 4 h, 200 µL FBS was added. At 24 h post-transfection, the medium was replaced with fresh medium, and incubation continued for an additional 24 h before harvesting for protein extraction.

### RT-PCR

Total RNA was isolated from cells using the NucleoSpin RNA kit. Reverse transcription of total RNA was performed using Maxima™ H Minus cDNA Synthesis Master Mix according to the manufacturer’s procedure. qPCR analysis was conducted using PowerUp SYBR Green Master Mix on a StepOnePlus Real-Time PCR System. The following primers were used for analysis:

NPRL2 (forward): CTGGTGTCCATCCTCCAGTACTCC

NPRL2 (reverse): ACTGGCCCTCTTGTGCCCTTG

NPRL3 (forward): TGCACGTCGGGCGTAGTTCG

NPRL3 (reverse): GCATGGTAGGGGCGGATGGC

DEPDC5 (forward): CAGCACAAGGAAACTACCTGGAG

DEPDC5 (reverse): CATGAGTAGGCGGTCCACTTCA

GAPDH (forward): CTGCACCACCAACTGCTTAG

GAPDH (reverse): AGGTCCACCACTGACACGTT

### Whole cell extract preparation and Western blotting

Cell pellets were washed with 1× PBS, resuspended in 100 μl NETN buffer supplemented with protease inhibitors, incubated on ice for 30 min, and sonicated at 30% intensity for 10–15 s. The samples were then centrifuged at 4 °C for 10 min at 12,000 rpm, and protein concentrations in each sample were determined with the BCA assay. 20 μg of total protein were mixed with LDS buffer containing 50 mM DTT, incubated at 90 °C for 10 min, centrifuged, and supernatant was collected. Equal amounts of total protein from each group of samples were resolved on 4−12% SDS–PAGE gels, and transferred onto a PVDF membrane, followed by incubation with 5% non-fat milk/TBST for 1 h. The membrane was then incubated with the primary antibody overnight at 4 °C, washed 3 times with TBST, and incubated with an HRP-conjugated secondary antibody in 5% non-fat milk/TBST for 1 h, washed 3 times with TBST, and developed with ECL reagent. The quantification of band intensities in the blot was performed using ImageJ software. The band intensity of the target protein was normalized to the band intensity of the indicated loading control protein.

### Detection of cisplatin–DNA adducts

2 × 10^6^ cells were seeded in T75 flasks. After overnight growth, cells were treated with 50 μΜ cisplatin for 24 h, trypsinized, centrifuged at 100×*g* for 5 min at 4 °C, and washed with PBS. The cells were then resuspended in 150 μl of MNase buffer (20 mM Tris–HCl, pH 7.5, 2.5 mM CaCl_2_, 5 mM NaCl), containing a protease inhibitor cocktail, and centrifuged at 16,000×*g* for 5 min. The supernatant (“soluble” fraction) was transferred to a separate tube. 10 U MNase in 100 μl MNase buffer was added to the pellet, incubated on ice for 10 min, centrifuged at 16,000×*g* at 4 °C for 5 min, and the supernatant (“MNase” fraction) was collected. 100 μl of 10× TBS buffer (1.5 M NaCl, 200 mM Tris base, pH 7.6) was added to the pellet, vortexed, and centrifuged for 5 min at 16,000×*g* at 4 °C. The supernatant (“High Salt” fraction) was collected and combined with the MNase fraction. 200 μl of 1× TBS buffer was added to the pellet, vortexed, and centrifuged for 5 min at 16,000×*g* at 4 °C. The supernatant was collected, combined with “High Salt + MNase” fraction, and referred to as the “chromatin” fraction. 2 μl RNAse A (10 mg/ml) was added to 150 μl of “chromatin” fraction and incubated for 1 h at 37 °C. 150 μl of Proteinase K 5 mg/ml in 1× Proteinase buffer was added to the “chromatin” fraction and incubated 1 h at 56 °C. 300 μl of Phenol:Chloroform:Isoamyl Alcohol (25:24:1) was added, vortexed, and centrifuged at 16,000×*g* for 5 min. The aqueous phase was transferred into a new tube, 1 volume of chloroform was added, samples were vortexed and centrifuged at 16,000×*g* for another 5 min. 10% v/v of 3 M sodium acetate and 3 volumes of cold ethanol were added to the aqueous phase, kept at −20 °C overnight, and spun at 16,000×*g* for 30 min at 4 °C. Ethanol was removed, pellet washed with 70% cold ethanol, dried at room temperature, and resuspended in water. 2 μl of 100 ng/μl DNA was loaded onto a Hybond membrane and allowed to dry. The membrane was placed for 1200 μs into the UV crosslinker (Stratalinker® UV Crosslinker 2400) set up at 254 nM and then incubated in SYBR Gold staining solution for 30 min. Excess of SYBR Gold was washed with 20% ethanol for 20 min, and the membrane was analyzed to identify the total amount of DNA. The same membrane was blocked with 5% milk in 1× TBST and incubated with anti-cisplatin modified DNA antibody (1:1000 dilution) overnight. The membrane was washed with TBST 3 times for 5 min each, incubated with anti-rat secondary antibody for 1 h, washed 3 times again for 5 min with TBST, and developed with ECL reagent.

### ICP-MS analysis of platinum accumulation

2 × 10^6^ cells were seeded in T75 flasks and maintained at 37 °C and 5% CO_2_ for 18–24 h, then 50 μM cisplatin was added for an additional 24 h. The cells were collected by trypsinization and counted. 1 × 10^6^ cells were centrifuged at 300×*g* for 5 min and washed twice with chilled 1× PBS. 200 μl of 70% HNO_3_ was added to the pellet, mixed gently, and incubated at 70 °C overnight. 100 μl of the resulting solution was mixed with 6900 μl of Milli-Q water to achieve the final HNO_3_ concentration of 1%. For accurate measurement, the cisplatin concentration should fall within a range between 50 ng/L and 10 μg/L. Metal concentrations were measured by ICP-MS [24] at the high-resolution analysis platform (PARI) at the Institut Physique du Globe de Paris.

### Immunofluorescence

Cells were seeded onto 18 mm coverslips placed in a 12-well plate. After 24 h, cells were treated or not with 50 μM cisplatin for 24 h. Cells were then fixed with 4% paraformaldehyde for 10 min at room temperature. Following three washes with 1× PBS, cells were permeabilized with 2% Triton X-100 for 10 min, followed by three washes with 1× PBS. After blocking with 0.5% BSA in 1X PBS for 40 min at room temperature, cells were incubated with rabbit polyclonal antibody against γH2AX (phospho-Ser139 H2AX, Active Motif, 39118, 1:500) for 2 h at room temperature, washed three times with 1× PBS and incubated for 1 h at room temperature with a secondary antibody conjugated to Alexa Fluor^TM^ 555 (Thermo Fisher Scientific, #A-21428, 1:200) followed by incubation with DAPI for 10 min. After washing with PBS, coverslips were mounted using a mounting medium. Images were acquired using a confocal SP8 microscope and were analyzed using ImageJ software.

### RNA-seq

The total RNA was isolated from cells using the NucleoSpin RNA kit according to the manufacturer’s instructions. RNA sequencing of three biological replicates for each sample was performed by BMKGENE (www.bmkgene.com) on the Illumina NovaSeq X platform, applying the Illumina PE150 strategy with 20 million reads per sample. Reads were mapped to the human reference genome (GRCh38.p10) and counted using HISAT2 and featureCounts. Before conducting the differentially expressed gene (DEG) analysis, low-expressed genes (where the number of samples with a count per million (cpm) greater than 1 was fewer than the number of samples in the comparison group with fewer sample arrays) were filtered out to reduce noise. Differential expression analysis was carried out with the R package DESeq2, and genes with an adjusted *p*-value < 0.01 and |log2-fold change| > 1 were considered differentially expressed. Gene Ontology (GO BP) and Kyoto Encyclopedia of Genes and Genomes (KEGG) databases were used for annotation, and Gene Set Enrichment Analysis (GSEA), were performed using the R package clusterProfiler. The principal component analysis (PCA) scatter plot was generated with factoextra and visualization—with ggplot2.

### Bioinformatics analysis

The original transcriptome data (from the TCGA–LUAD, TCGA-LUSC projects, as well as GTEX), clinical information, and a human genome reference document for ENSG number annotation were downloaded from the open-access UCSC Xena database (https://xenabrowser.net/datapages/). After duplicating and annotating the genes, the expression matrix for TCGA-LUAD, TCGA-LUSC, and GTEX was obtained for drug sensitivity prediction. Data on the chemotherapeutic response to cisplatin and RMA-normalized expression data for cell lines were downloaded from the largest available public pharmacogenomics database, GDSC (https://www.cancerrxgene.org/downloads/bulk_download). The prediction process was completed using the R package “oncoPredict” for drug cisplatin sensitivity in LUAD and LUSC patients. The correlation between the GATOR1 complex and apoptosis pathway scores was analyzed by Spearman correlation, and a correlation map was generated by using the R package “ggplot2”.

## Results

### Deletion of GATOR1 components renders cells resistant to cisplatin

**Fig. 1:**
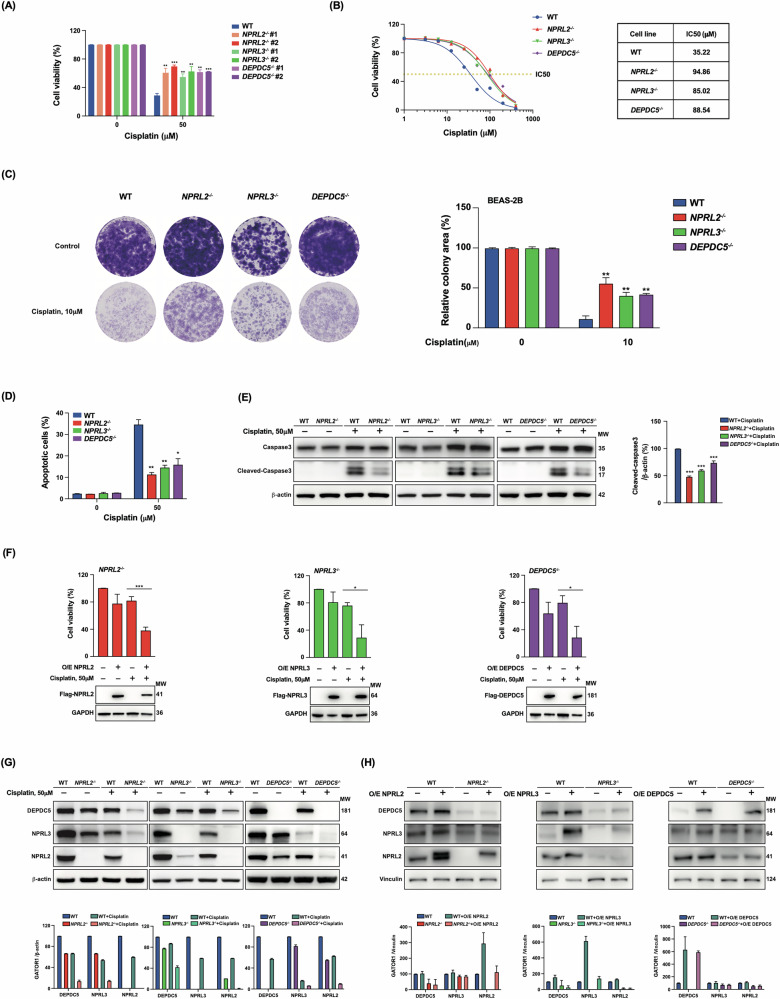
BEAS-2B cells with deletions of GATOR1 components are resistant to cisplatin. **A** Cell viability test by the MTT assay of two clones for each homozygous deletion of the indicated GATOR1 components. Cells were treated with 50 μM cisplatin for 24 h. **B** Identification of IC_50_ in clone #2 for each GATOR1 deletion in cells treated with different cisplatin concentrations by the MTT test, as in (**A**). **C** Cell proliferation assay in the indicated BEAS-2B cell lines. Note that for this assay, cells were treated with 10 μM cisplatin, since 50 μM cisplatin was too high in this setting. See the “Materials and methods” section for details. **D** Analysis of apoptosis by Annexin V/7-AAD assay and caspase 3 cleavage (**E**). **F** MTT analysis of cisplatin sensitivity in GATOR1 deletion cells transfected with the indicated plasmids for 36 h. Representative Western blot images from three independent experiments are shown in (**B**, **E**, and **F**). **G** Western blot analysis of the expression of different GATOR1 components in the indicated deletion cell lines, with or without cisplatin treatment (50 μM, 24 h). **H** Western blot analysis of expression of GATOR1 components when one of the members is overexpressed.

A correlation between low expression of NPRL2 and cisplatin resistance has been previously reported for various non-small-cell lung cancer cell lines [20, 25]. To investigate the reasons for this resistance, we analyzed several NSCLC cell lines as well as BEAS-2B, an immortalized but non-cancerous cell line derived from human bronchial epithelium [26]. We separately deleted each of the GATOR1 components in BEAS-2B cells using CRISPR-Cas9 technology. The cisplatin sensitivity of cells with homozygous deletions was evaluated by MTT in two clones for each deletion (Fig. 1A). As both clones showed comparable viability, we proceeded with clone #2 for each respective deletion.

The MTT analysis of cell viability indicated that resistance to cisplatin was increased not only after NPRL2 deletion but also after deletion of two other GATOR1 components (Fig. 1A, B). Similar results were obtained in HEK293T cells with GATOR1 deletions (Fig. S1A). The IC_50_ of GATOR1-deleted BEAS-2B cell lines was ~90 μM, while the IC_50_ of wild-type cells was around 35 μM (Fig. 1B). The colony formation assay also showed that BEAS-2B cells with GATOR1 deletions better resisted cisplatin treatment (Fig. 1C).

Incubation with the drug induced less apoptosis in GATOR1-deleted BEAS-2B cells compared to wild type, as determined by Annexin V/7-AAD assay (Fig. 1D). Impaired apoptosis was validated by reduced caspase 3 cleavage in GATOR1 deleted cells after cisplatin treatment (Fig. 1E). To confirm that observed effects are specific to GATOR1 deletions and were not artifacts of CRISPR/Cas9 manipulations, we overexpressed the corresponding GATOR1 component from a CMV promotor-driven plasmid in the respective knockout cells and observed the restoration of drug sensitivity (Fig. 1F).

We also observed that deletion of any GATOR1 member in both BEAS-2B and HEK293T cells led to decreased expression of the other two components, an effect particularly pronounced in BEAS-2B cells following cisplatin treatment (Fig. 1G, Fig. S1B). However, overexpression of a single GATOR1 subunit did not affect the expression of the other two components in wild-type cells and, importantly, was unable to restore their expression in the respective deletion cell lines (Fig. 1H). These observations indicate that GATOR1 stability depends on correct stoichiometry and inter-subunit interactions.

### GATOR1 overexpression induces cisplatin sensitivity in NSCLC-resistant cells

**Fig. 2:**
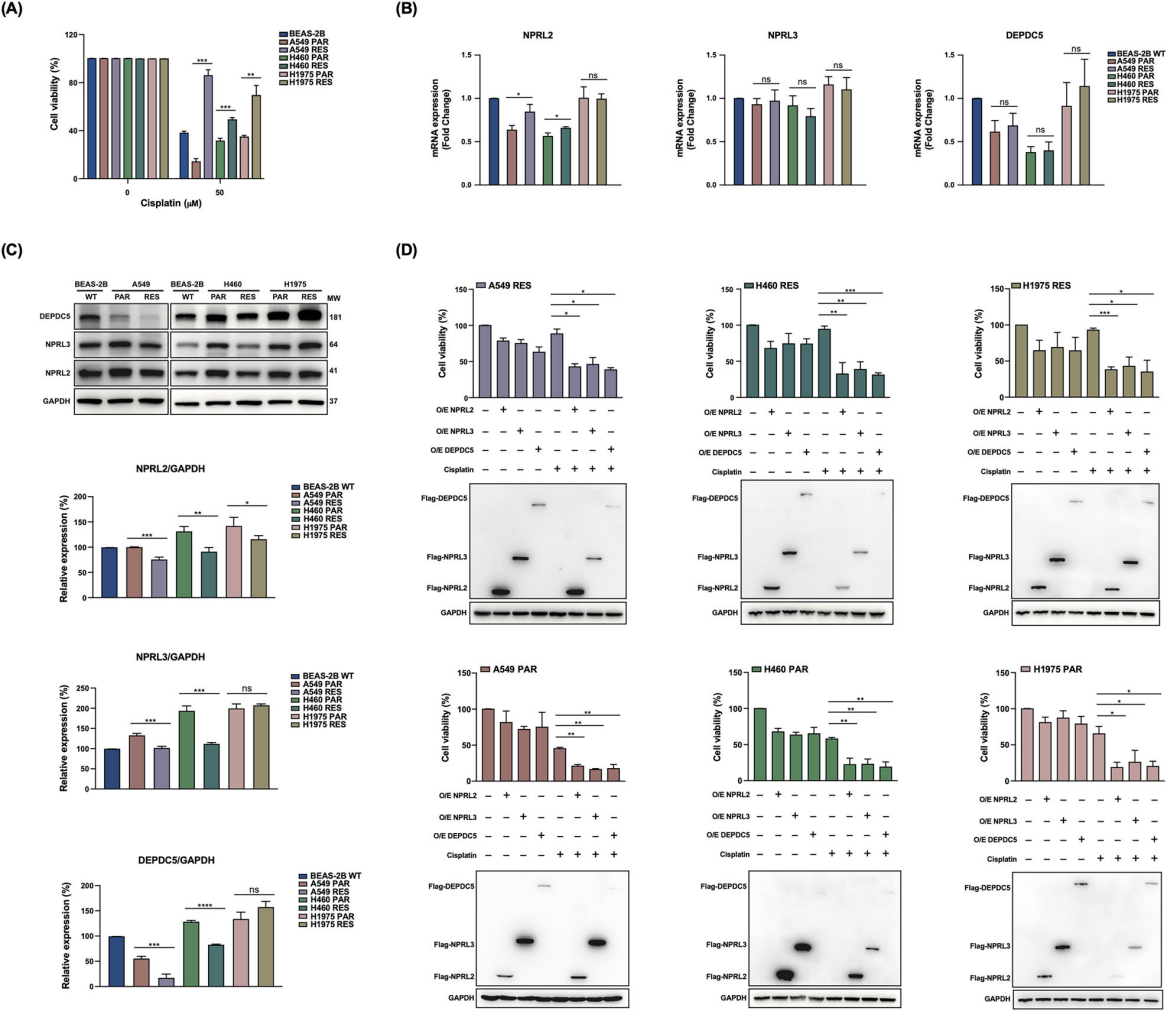
GATOR1 components overexpression increases cisplatin sensitivity in cancer cells. **A** Cell viability of indicated parental (PAR) and cisplatin-resistant (RES) NSCLC cell lines compared to BEAS-2B wild-type cells was evaluated by the MTT assay. **B** mRNA expression levels of the three GATOR1 members in the indicated NSCLC cell lines compared to BEAS-2B wild-type cells. **C** Protein expression of GATOR1 components in the indicated cell lines. MW molecular weight of probed proteins. A representative western blot image from three independent experiments is shown. The relative expression of each protein was normalized to GAPDH after calculation of band intensities by Image J. **D** FLAG-tagged NPRL2, NPRL3, or DEPDC5 were overexpressed in the indicated NSCLC cell lines for 36 h and then treated with 50 μM cisplatin for 24 h. An MTT assay was used to detect cell sensitivity to cisplatin.

We next examined the status of GATOR1 expression in cisplatin-sensitive and resistant H460, H1975, and A549 NSCLC cell lines (Fig. 2). H460 cells harbor the KRAS^Q61H^ mutation and are p53 wild-type; H1975 cells carry the EGFR^L858R,T790M^ mutations along with TP53^R273H^ mutation; A549 cells have a KRAS mutation but EGFR and TP53 wild type. The resistant derivatives were obtained by escalating doses of cisplatin, which can be considered as a model of cells with acquired cisplatin resistance [15, 23].

We confirmed the sensitivity of these cells to the drug using an MTT assay (Fig. 2A). We also performed a colony formation assay for A549 and A549res cells (Fig. S1C), which confirmed the increased resistance of A549 to cisplatin. The similar results for H460 and H1975 sensitive and resistant cells were published previously [15]. We further analyzed mRNA and protein expression of GATOR1 members in these cells. We found that compared to BEAS-2B cells, mRNA levels of NPRL2 and DEPDC5 were significantly decreased both in wild-type and resistant H460 and A549 cells, but did not change in H1975 cells, while NPRL3 levels slightly decreased only in H460res cells (Fig. 2B). Otherwise, there were no remarkable differences between mRNA levels in sensitive and resistant cells.

The protein levels of any GATOR1 component were lower in H460res and A549res cells but did not significantly change in sensitive and resistant H1975 cells (Fig. 2C). Similarly, as observed in non-cancerous cells (Fig. 1D), overexpression of any of GATOR1 component slightly reduced cell viability in all types of untreated NSCLC cells and significantly, especially in resistant cells, when cisplatin was added (Fig. 2D). Thus, restoration and/or overexpression of GATOR1 enhances cisplatin sensitivity not only in non-tumor cells but also in NSCLC cells.

### Upregulation of mTORC1 activity correlates with cisplatin resistance

**Fig. 3:**
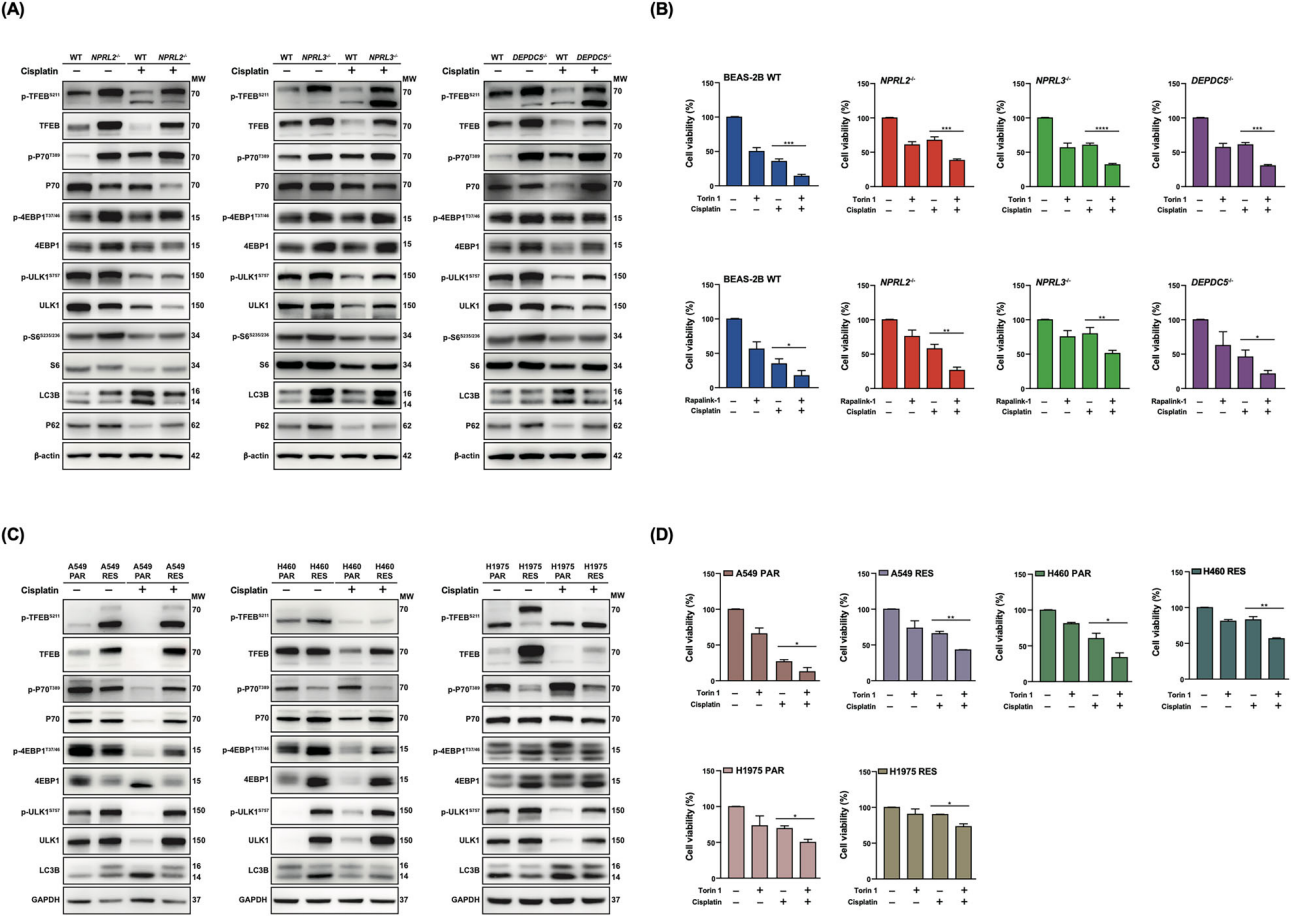
mTORC1 activity and autophagy are compromised in BEAS-2B cells with GATOR1 deletions and NSCLC cells. **A** Analysis of mTORC1 effectors expression by western blot in the indicated BEAS-2B cells with GATOR1 deletions, treated or not with cisplatin. MW—molecular weight of probed proteins. **B** The indicated BEAS-2B cells were pre-treated with 1 μM of Torin 1 for 2 h (upper panel) or 10 nM of RapaLink-1 for 4 h (lower panel). 50 μM cisplatin was added where indicated and incubated for 24 h. Cell viability was evaluated by MTT assay. **C** and **D** Analysis of mTORC1 activity and autophagy in parental and resistant NSCLC cells, as in (**A**) and (**B**). Representative western blot images and MTT results from three independent experiments are shown.

Because mTORC1 activity is often changed in cisplatin-resistant cells [11, 15], we further analyzed the phosphorylation status of the four most prominent mTORC1 targets in GATOR1-deleted cells (4E-BP1, TFEB, ULK1, and p70) as well as the p70 target S6, with or without drug treatment (Fig. 3A). Phosphorylation of two of these targets, 4E-BP1 and p70, promotes anabolism. i.e., synthesis of nucleotides, proteins, and lipids, while phosphorylation of the other two—the autophagy initiation component ULK1 and the transcription factor EB (TFEB)—inhibits catabolic pathways.

As expected, deletion of GATOR1 components increases the phosphorylation of selected mTORC1 substrates. However, these substrates did not behave similarly after cisplatin treatment. For example, in wild-type cells, the addition of the drug reduced the total level and phosphorylation of ULK1 and TFEB (Fig. 3A). Simultaneously, phosphorylation of p70 was strongly induced, but there was no significant change for 4E-BP1. In GATOR1-deleted cells, the phosphorylation level remained practically unchanged (and was more elevated compared to wild-type cells) for all the substrates after cisplatin treatment, except for ULK1, which decreased in both total and phosphorylated amounts.

Because phosphorylation of ULK1 by mTORC1 suppresses autophagy, we also assessed the autophagic status of BEAS-2B wild-type and GATOR1-deleted cells before and after cisplatin treatment by evaluating the expression and lipidation of LC3B as well as the expression of p62 (Fig. 3A). Here again, we observed differences between GATO R1 components. For example, DEPDC5 deletion did not have a significant impact on LC3B expression and lipidation, while NPRL2 deletion suppressed basal autophagy. In wild-type cells, as expected, cisplatin treatment slightly enhanced LC3 expression, but no significant changes in LC3 expression and lipidation were observed in GATOR1-deleted cells. The analysis of p62 expression demonstrated that it was increased in all deletion cells in comparison to wild-type cells, with or without drug treatment. p62 is a selective autophagy adapter that is normally degraded via autophagy. Hyperactive mTORC1 in GATOR1 KO cells inhibits autophagy, leading to p62 accumulation. Therefore, the increased p62 levels observed in GATOR1 KO cells reflect impaired autophagy downstream of mTORC1 hyperactivation. Concurrent treatment of wild-type and mutant BEAS-2B cells with the dual mTORC1/mTORC2 inhibitor Torin 1 and cisplatin decreased cell viability, yet mutant cells survived better than wild-type cells (Fig. 3B, upper panel). We also repeated these experiments with the highly specific mTORC1 inhibitor RapaLink-1 (Fig. 3B, lower panel). The results obtained with RapaLink-1 closely mirrored those with Torin1 (Fig. 3B), indicating that the observed effect is attributable to mTORC1 inhibition rather than mTORC2 suppression.

mTORC1 activity in NSCLC-resistant and sensitive cells was addressed previously [15, 27], although only a limited set of mTORC1 effectors was analyzed. Given the differences observed in BEAS-2B cells with various GATOR1 deletions, we examined the status of four mTORC1 effectors in NSCLC parental and resistant cells. Phosphorylation of all tested mTORC1 targets was significantly upregulated in resistant cells compared to parental cells, except for p70 in H460 and H1975 cells (Fig. 3C). However, in contrast to BEAS-2B cells with GATOR1 deletions, cisplatin treatment had no effect on mTORC1 activity in NSCLC cells, which remained elevated. NSCLC parental cells, especially A549, exhibited higher levels of autophagy compared to their resistant counterparts, consistent with previous findings [14, 15]. Treatment with cisplatin enhanced autophagy in parental cells but did not induce autophagy in resistant cells. (Fig. 3C). Similar to BEAS-2B cells with GATOR1 deletions, despite the enhanced resensitization of NSCLC cells to cisplatin observed with the combined treatment of cisplatin and Torin 1 compared to treatment with the drug alone (Fig. 3D), resistant cells still exhibited greater resistance to the drug than parental cells. Therefore, while cisplatin-resistant cells exhibit heightened mTORC1 activity, it is evident that hyperactivation of this metabolic pathway is just one factor contributing to drug resistance.

### GATOR1 deletion results in the formation of less DNA–cisplatin adducts and DNA damage, but induces more DNA damage response

**Fig. 4:**
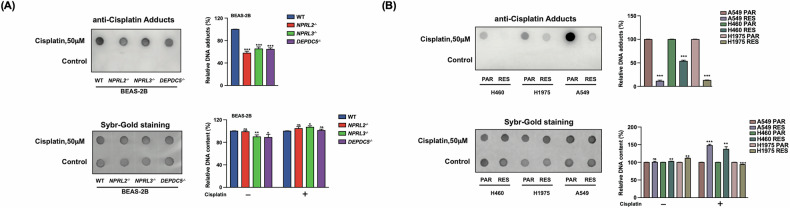
Analysis of cisplatin-DNA adducts. **A** Dot-blot with an antibody recognizing cisplatin-modified DNA to measure the accumulation of cisplatin–DNA adducts (graph) after treatment of indicated BEAS-2B cell lines with cisplatin (50 μM, 24 h). SYBR Gold staining was used as a loading control. **B** The same analysis as in (**A**) for the indicated parental (PAR) and cisplatin-resistant (RES) NSCLC lines.

After establishing that GATOR1 mutants exhibit metabolic characteristics resembling cisplatin-resistant cells, we began investigating the reasons behind this drug resistance. One potential explanation is the formation of fewer DNA–cisplatin adducts. DNA is the primary target of cisplatin, and if fewer crosslinks are formed between the drug and its target, less damage would be induced, allowing cells to better resist treatment. Indeed, we detected 30–40% fewer DNA–cisplatin adducts in GATOR1-deleted cells (Fig. 4A).

In all NSCLC resistant cells tested, we also observed a reduction in adducts, but to a more drastic extent compared to GATOR1 mutants—up to 80–90% (Fig. 4B). This is not surprising, given that these cells were selected after several weeks of cultivation with increasing concentration of cisplatin, reaching a final concentration of 2000 ng/ml for A549res cells [23] and in the range between 5 nM and 2.5 μM for H460res and H1975res cells [15].

**Fig. 5:**
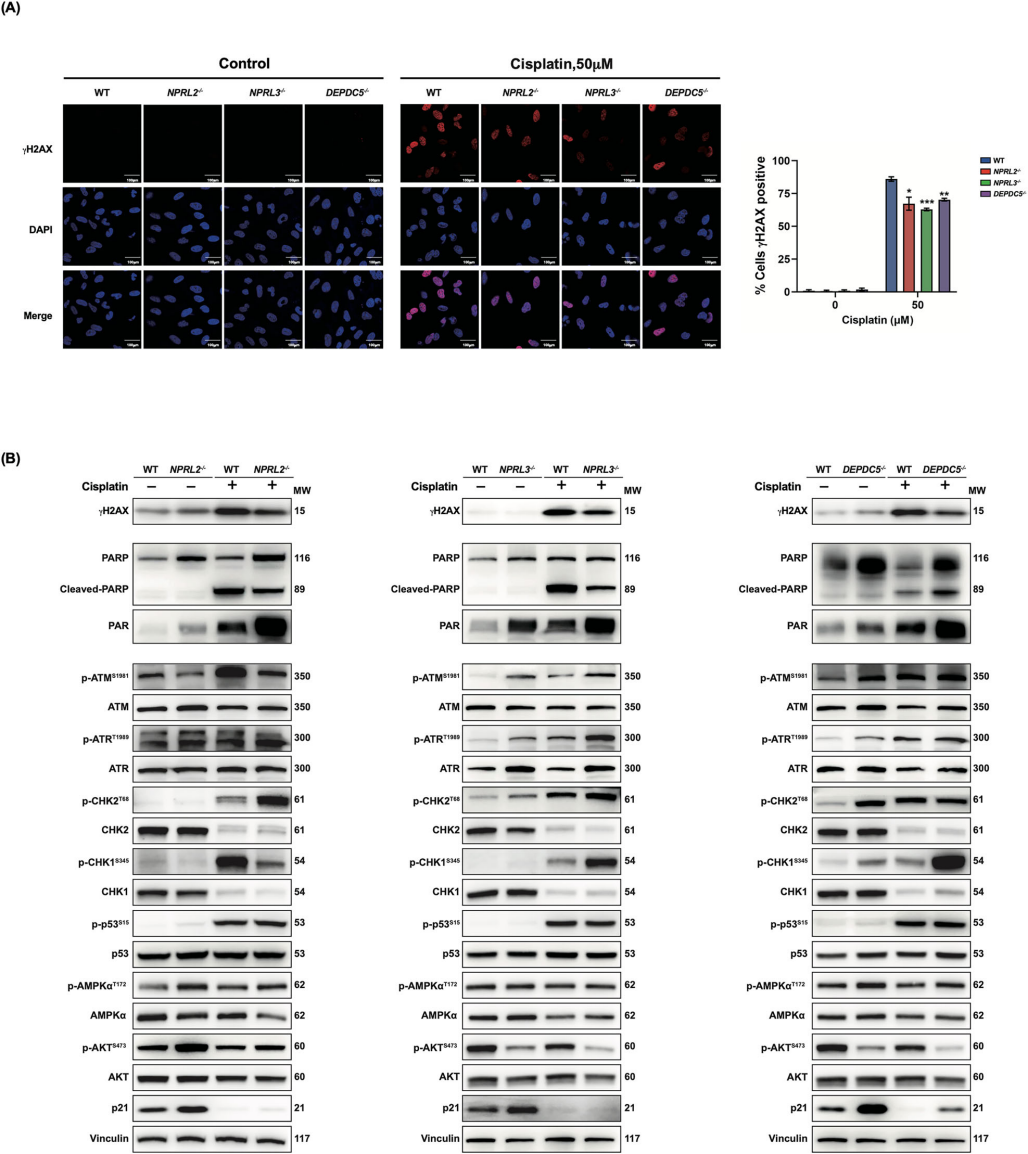
Analysis of DNA damage response. **A** Immunofluorescence analysis of γH2AX in BEAS-2B WT, NPRL2^−/−^, NPRL3^−/−^, and DEPDC5^−/−^ cells, either untreated (NT) or treated with 50 µM cisplatin for 24 h. (left) Representative confocal images showing γH2AX staining (red) and nuclear stained with DAPI (blue). Scale bars: 100 µm. (right) Quantification of the percentage of γH2AX-positive foci. Data represent mean ± SEM from three independent experiments (100–150 cells counted per condition). Statistical significance was determined by unpaired two-tailed Student’s *t*-test: *P < 0.05, **P < 0.01, ***P < 0.001. **B** BEAS-2B wild-type cells and cells with GATOR1 deletions were probed with the indicated antibodies to evaluate the status of DNA damage response sensors and effectors with or without cisplatin treatment. MW molecular weight of probed proteins. Representative western blot images from three independent experiments are shown.

We next verified the ability of cisplatin to induce DNA damage and trigger the DNA damage response in GATOR1 mutants. Histone H2AX phosphorylated at Ser139 (γH2AX) serves as a highly specific and sensitive molecular marker for DNA damage. As shown in Fig. 5A, after cisplatin treatment, the number of γH2AX-positive foci was greatly increased, but was lower in the deletion cells in comparison to wild-type cells. The same result was obtained by Western blot (Fig. 5B). Indeed, the expression level increased in both wild-type and mutant BEAS-2B cells. However, in cells with GATOR1 deletion, the expression level of γH2AX was significantly lower compared to wild-type cells, indicating that less DNA damage was induced in GATOR-deleted cells.

We further examined the expression of DNA damage sensors ATM and ATR kinases, involved in γH2AX phosphorylation, as well as the expression of poly(ADP-ribose) polymerase 1 (PARP1). After DNA damage, PARP1 is recruited to DNA breaks, whereupon activation, it catalyzes poly(ADP-ribosyl)ation (PARylation) of itself and many other proteins. Cells with GATOR1 deletions exhibited both PARP1 overexpression and high abundance of PAR-containing proteins, which were significantly increased in *NPRL2*^−/−^ and *DEPDC5*^−/−^ cells after drug treatment, as compared to wild-type cells. Although *NPRL3*^−/−^ cells did not show PARP1 overexpression, they still exhibited high levels of PARylation under the tested conditions, suggesting changes in PARylation/dePARylation homeostasis in those cells. This parallels observations of high PARP1 expression and high PARylation levels in various cisplatin-resistant NSCLC cell lines, including A549 [28, 29].

Activation of ATM and ATR is linked to their autophosphorylation at S1981 for ATM and T1989 for ATR. Once activated, ATM phosphorylates and activates CHK2, p53, and, to a lesser extent, CHK1, while ATR primarily targets CHK1. These events lead to the stimulation of p21 transcription, resulting in G1 arrest in cells with active p53 and S or G2/M arrest in cells with inactive p53 [21]. We verified most components of this phosphorylation cascade in BEAS-2B cells and found here again that each GATOR1 mutant exhibited slightly different behavior. For example, ATM phosphorylation was decreased in *NPRL2*^−/−^ cells compared to wild-type cells both before and after drug treatment, whereas it increased in *NPRL3*^−/−^ and *DEPDC5*^−/−^ cells and did not change further upon cisplatin treatment. Phosphorylation of CHK2 at T68 was more abundant in *NPRL2*^−/−^ cells, while phosphorylation of CHK1 at S345 was increased more in *DEPDC5*^−/−^ cells. The expression and phosphorylation of p53 were similar in the wild type and all three mutant cells. One of the targets of p53 is the AMPKα subunit, which is phosphorylated at Thr172 by LKB1 during DNA damage, leading to inhibition of mTORC1. We did not detect significant differences between wild-type and GATOR1 mutants concerning total AMPKα levels and their phosphorylation. Finally, p21 was increased in all three mutants, especially in *DEPDC5*^−/−^ cells.

AKT kinase is another downstream target of ATM and ATR during the DNA damage response and is also a well-known upstream regulator of mTORC1 [12]. Phosphorylation of AKT at Ser473 by ATM and ATR results in its activation and upregulation of mTORC1 activity. However, mTORC1-promoted p70 phosphorylation may lead to decreased AKT activity via initiation of a major negative feedback loop upstream of AKT. Surprisingly, phosphorylation of AKT at Ser473 was decreased in *NPRL3*^−/−^ and *DEPDC5*^−/−^ cells compared to wild-type BEAS-2B, but upregulated in *NPRL2*^−/−^ cells, probably indicating a fine-tuning of mTORC1 activity by different components of GATOR1.

In summary, GATOR1-deleted BEAS-2B cells form fewer DNA adducts with cisplatin and exhibit less DNA damage. At the same time, the DNA damage response is more active in these cells.

### Cells with GATOR1 deletions accumulate less cisplatin due to altered expression of its influx and efflux transporters

**Fig. 6:**
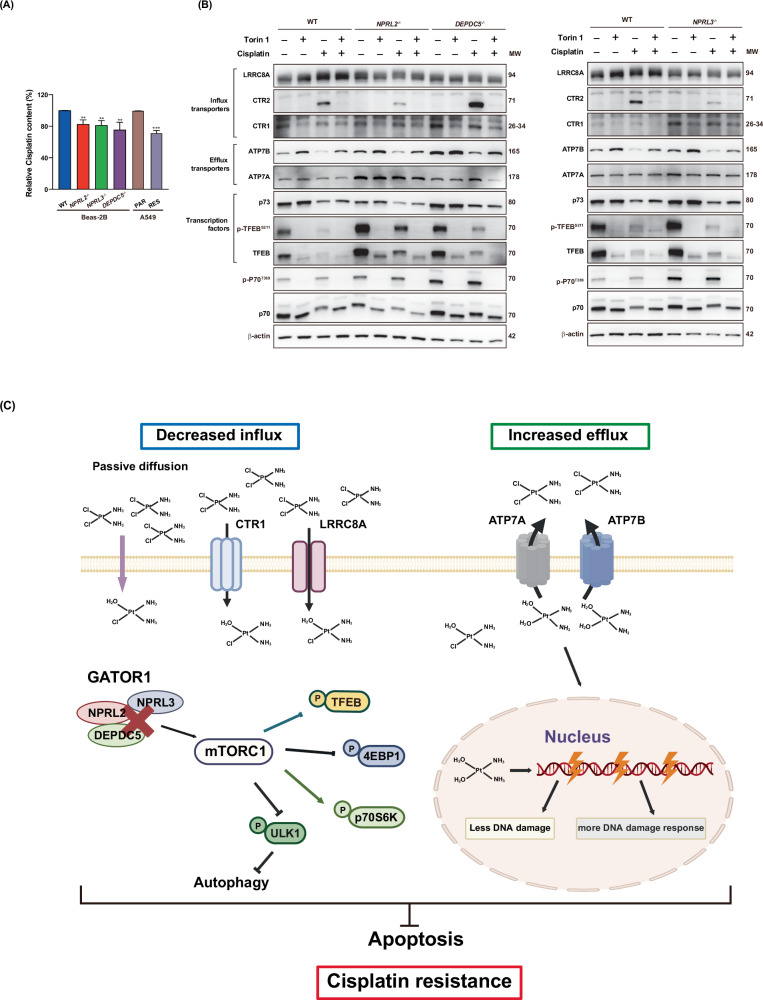
Analysis of cisplatin accumulation and expression of cisplatin efflux and influx transporters. **A** The indicated BEAS-2B and A549 cells were treated with cisplatin and subjected to an ICP-MS assay to test cisplatin accumulation. The graph demonstrates the relative cisplatin content in equivalent amounts of cells. **B** Expression of efflux and influx transporters was tested by western blot in the indicated cells treated or not with cisplatin (50 μM, 24 h), Torin 1 (1 μM, 2 h), or a combination of drugs (treatment with Torin 1 for 2 h, then cisplatin addition for 24 h). MW molecular weight of probed proteins. Representative western blot images from three independent experiments are shown. **C** Schematic representation of the role of GATOR1 in cisplatin resistance. Deletion of GATOR1 components increases mTORC1 activity and expression of efflux transporters and decreases expression of influx transporters. This results in decreased accumulation of the drug in the cells and milder DNA damage. In contrast, the DNA damage response is enhanced in these cells.

We further hypothesized that the decreased formation of cisplatin-DNA adducts and subsequent diminished DNA damage in GATOR1 mutants might result from reduced drug accumulation in these cells. To investigate this, we measured the cellular accumulation of cisplatin using inductively coupled plasma mass spectrometry (ICP-MS). The results revealed that GATOR1-deleted cells contained approximately 20–25% less drug compared to the wild type. This reduction was similar to that observed in A549 cells, where the resistant counterparts accumulated ~30% less cisplatin (Fig. 6A).

The main reasons for decreased cisplatin accumulation are impaired influx and/or efflux of the drug. Cisplatin can enter cells either by passive diffusion or via active influx through copper transporters (CTR1, CTR2) [30] or via a volume-regulated anion channel composed of LRRC8A and LRRC8D [5, 31]. The efflux of the drug can be modulated by copper efflux transporters ATP7A and ATP7B [32], both being transcriptional targets of TFEB [33, 34]. TFEB continuously shuttles between the nucleus and the cytoplasm. Nutrient availability triggers the nuclear export and cytoplasmic inactivation of TFEB through phosphorylation by mTORC1 at multiple sites [35]. Cisplatin treatment causes the nuclear translocation of TFEB, at least in ovarian cancer cell lines [36]. In addition, ATP7A is also under the control of p73 transcription factor [37].

We assessed the expression of all these transporters in the wild-type BEAS-2B cells and GATOR1-deleted cells treated or not with cisplatin. We also treated cells with Torin1 separately or together with cisplatin to evaluate if changes in expression depend on upregulation of mTORC1 activity in GATOR1-deleted cells.

The expression of the LRRC8A influx transporter was downregulated in GATOR1 mutants with or without treatment. The addition of Torin1 did not have a significant effect on LRRC8A expression. CTR2 expression significantly increased in wild-type cells after incubation with cisplatin, and even more in *DEPDC5*^−/−^ cells, but decreased in *NPRL2*^−/−^ and *NPRL3*^−/−^ cells. As for CTR1, its expression in all three GATOR1-deleted cells was comparable with that of wild-type cells and did not change significantly after treatment with any of the drugs or their combination.

Interestingly, ATP7B expression was decreased in both wild-type and mutant cells treated with cisplatin, but enhanced in the cells treated with Torin1 or both Torin1 and cisplatin. As for ATP7A, its expression was increased in all GATOR1-deleted cells compared to wild-type cells after cisplatin treatment (Fig. 6B). This might be due to the enhanced transcription of ATP7A by TFEB or by p73. p73 level was decreased in all cells treated with cisplatin or in the cells treated with both Torin1 and cisplatin. Deletion of GATOR1 components not only enhances TFEB phosphorylation but also significantly increases the total amount of TFEB (Fig. 3A). Both total and phosphorylated TFEB levels are also elevated in NSCLC cisplatin-resistant cells (Fig. 3C). Thus, despite the elevated mTORC1 activity and increased phosphorylation of TFEB in cisplatin-resistant cells, a substantial portion of the transcription factor can still be present in the nucleus. This nuclear presence can be sufficient to maintain elevated transcription of cisplatin efflux transporters, sustaining drug resistance.

As expected, TFEB phosphorylation was completely abolished by Torin 1 treatment in both wild-type and GATOR1-deleted cells. Cisplatin treatment alone significantly reduced expression of TFEB, which nevertheless remained almost entirely phosphorylated. Treatment with both drugs had a synergetic effect, further reducing the expression of total TFEB while abolishing its phosphorylation. Importantly, expression of unphosphorylated TFEB remains elevated in GATOR1-deleted cells, indicating that it does not depend on mTORC1. Surprisingly, ATP7A expression did not change significantly after treatment with either drug or their combination and remained constitutively elevated in cells with NPRL2 and DEPDC5 deletions with or without treatment (cells with NPRL3 deletions were comparable with the wild type), suggesting that enhanced ATP7A expression in these cells does not depend on p73, mTORC1, and TFEB.

Thus, cisplatin resistance in GATOR1-deleted BEAS-2B cells may be attributed, at least in part, to diminished cisplatin concentration resulting from impaired influx and enhanced efflux of the drug (Fig. 6C).

### Transcriptomics analysis of cells with GATOR1 deletion reveals global changes in the transcriptional and metabolic programs in relation with cisplatin resistance

**Fig. 7:**
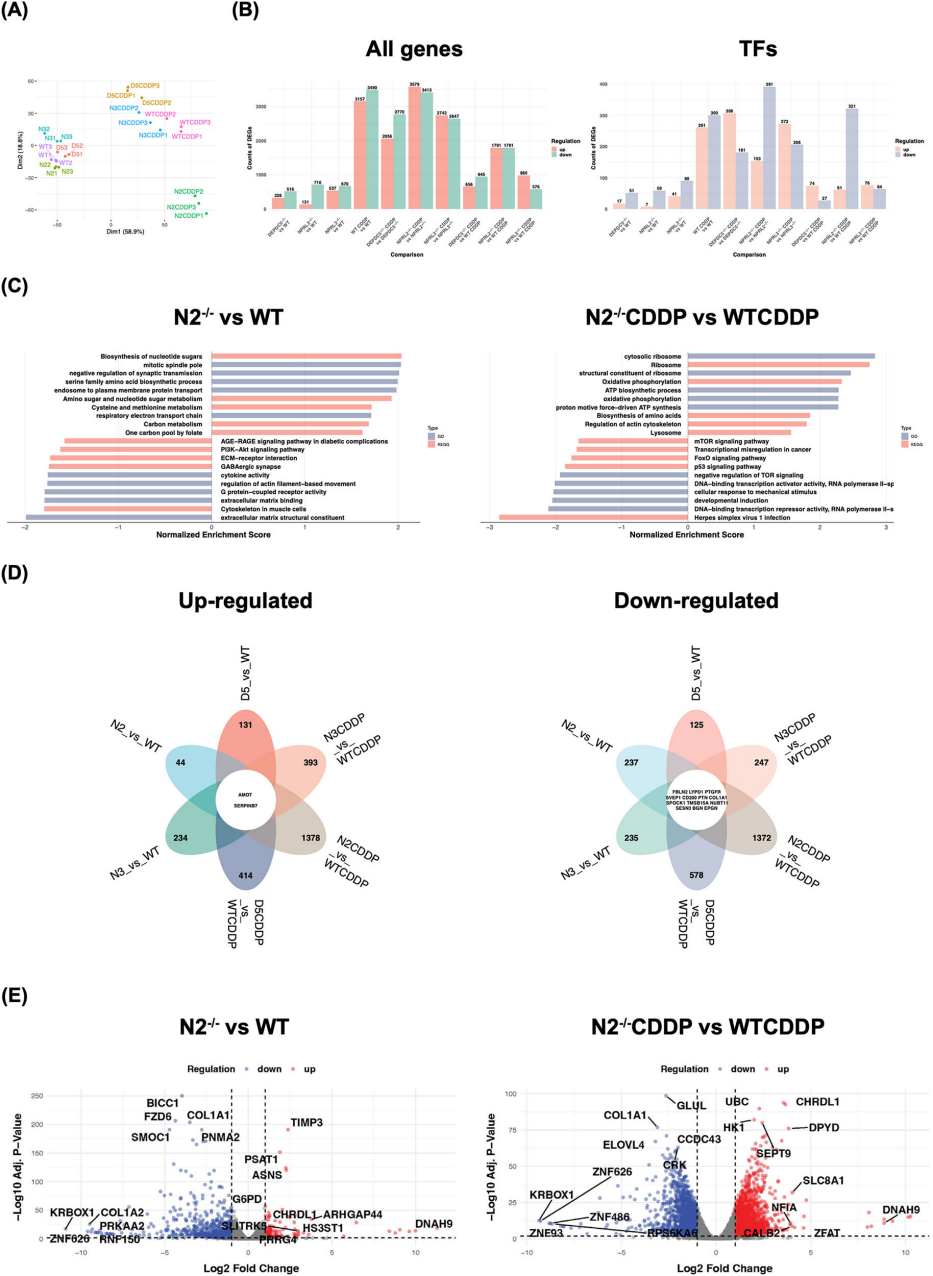
Analysis of RNA-seq data from BEAS-2B cells with GATOR1 deletions. **A** A principal component analysis (PCA) plot illustrating the clustering of samples based on gene expression data, with different colors representing samples from different groups. PCA analysis plot displays 24 samples along principal components 1 and 2 (Dimensions 1 and 2 (Dim1 and Dim2)), which describe 18.8% and 58.9% of the variability, respectively, within the expression data set. WT1, WT2, and WT3 denote the three independent replicates of the wild-type group; the same applies to the other groups. N2—*NPRL2*^−/−^, N3—*NPRL3*^−/−^, D5—*DEPDC5*^−/−^, CDDP—cisplatin-treated. **B** A bar graph displaying the differentially expressed genes (DEGs) and the number of differentially expressed transcription factors (TFs) obtained from pairwise comparisons of each GATOR1 knockout group relative to the wild-type (WT) group. **C** Bar graphs of GSEA analysis results for the *NPRL2*^−/−^ group compared to the WT group (left) and the same pair of groups treated with cisplatin (right). The *x*-axis represents the normalized enrichment score, with blue indicating GO pathways and red indicating KEGG pathways. **D** A Venn diagram showing the intersection of DEGs derived from pairwise comparisons between the WT group and each GATOR1 knockout group (up-regulated genes are on the left, down-regulated genes are on the right). Each circle represents a group-pair in differential expression analysis. The intersection of circles indicates common DEGs between group pairs. The central circle displays gene symbols that are significantly up-regulated (two genes) or down-regulated (13 genes) in the GATOR1 knockout groups compared to the WT group across all selected pairwise comparisons. The numbers in the petals indicate the quantity of unique DEGs in each pairwise comparison. **E** Volcano plots of DEG analysis results for the NPRL2^−/−^ group compared to the WT group (left) and the same pair of groups treated with cisplatin (right). The *x*-axis represents Log2(Fold Change), while the *y*-axis represents −Log10(Adjusted *p*-value). The two dashed lines indicate the thresholds for filtering DEGs with Adjusted *p*-value < 0.01 and |Log2(Fold Change)| > 1. Red points represent up-regulated genes, blue points represent down-regulated genes, and gray points indicate genes with no significant differences. Among the up/down-regulated genes, the top 5 gene symbols with the highest −Log10(Adjusted *P*-Value) and |Log2(Fold Change)| are marked on the plot.

In order to expand our understanding about overall changes in BEAS-2B cells following GATOR1 deletion and the perturbations induced by cisplatin treatment, we conducted RNA sequencing of these cells treated or not with the drug and compared gene expression with wild type cells (Figs. 7, S2–S5, Tables S1–S3).

To evaluate global expression patterns and explore the relationships among samples, principal component analysis (PCA) was performed (Fig. 7A). The first two principal components (PC1 and PC2 or Dim1 and Dim2) explained 58.9% and 18.8% of the total variance, respectively, indicating that the main differences among samples were effectively captured. Samples belonging to the same experimental group clustered closely together, demonstrating high intra-group consistency (Fig. 7A). The distinction between cisplatin-treated and untreated groups was significantly more pronounced than that between GATOR1-deleted and wild-type groups, suggesting that cisplatin treatment induces more substantial transcriptomic and biological alterations in BEAS-2B cells compared to GATOR1 deletion (Fig. 7A). Notably, *NPRL2*^−/−^ samples displayed differences along PC2 in relation to WT, while *NPRL3*^−/−^ and *DEPDC5*^−/−^ samples exhibited contrasting differences along PC2 compared to WT (Fig. 7A). These variations were further amplified by cisplatin treatment, indicating that, despite all being components of the same complex, NPRL2 may have distinct roles in specific biological processes compared to DEPDC5 and NPRL3.

Various pairwise comparisons revealed numerous differentially expressed genes (DEGs), with the number of DEGs between the cisplatin-treated and untreated groups surpassed 5000, indicating that cisplatin profoundly influences gene expression profiles and biological processes in BEAS-2B cells (Fig. 7B). Without drug treatment, the amount of DEGs is similar for deletion of each GATOR1 component compared to wild type cells (842 for *DEPDC5*^−/−^, 841 for *NPRL2*^−/−^, and 1207 for *NPRL3*^−/−^), with the majority of genes being downregulated. However, there is a notable difference in the ratio of down- and upregulated genes. Specifically, in *NPRL2*^−/−^ cells compared to wild type, there are ~5 times more downregulated genes (84%) than upregulated (710 vs. 131), which may be attributed to the fact that there are ~8 times more downregulated than upregulated transcription factors (Table S1). In *NPRL3*^−/−^ cells, 55% of genes are, and in *DEPDC5*^−/−^ cells, 60% are downregulated.

Treatment with cisplatin significantly increased the number of differentially expressed genes: 30% more for *NPRL3*^−/−^, twice as many for *DEPDC5*^−/−^, and four times as many for *NPRL2*^−/−^ (Fig. 7B). The ratio between upregulated and downregulated genes did not change for *DEPDC5*^−/−^, but reversed for *NPRL3*^−/−^ (40% downregulated) and became equal for *NPRL2*^−/−^. Interestingly, these changes were not always correlated with alternation in differentially expressed transcription factors. For example, in *NPRL2*^−/−^ cells, the percentage of upregulated transcription factors was practically the same as in the cisplatin-treated cells (11% vs. 14%), while the percentage of upregulated genes increased threefold, from 16% to 50% after incubation with the drug. In contrast, the percentage of upregulated genes did not change in *DEPDC5*^−/−^ cells after cisplatin treatment, while the proportion of upregulated transcription factors increased threefold (from 25% to 75%) (Table S1). The most deregulated transcription factors belonged to the groups of bHLH, Forkhead, Homebox, TF-bZIP, ZBTB, and zf-C2H2 (Table S2). Some of these factors, such as AR, TFEB, and TFE3, are direct targets of mTORC1 [38].

Gene set enrichment analysis (GSEA) identified multiple significantly enriched GO and KEGG signaling pathways. The pathways with the highest absolute normalized enrichment scores were, as expected, predominantly related to metabolism, signal transduction and regulation (including PI3K-AKT and mTOR pathways), as well as extracellular matrix, cytoskeletal functions, and herpes simplex virus 1 infection (Fig. 7C, S2). For example, in *NPRL2*^−/−^ cells, seven out of the ten most enriched pathways are related to metabolism. Additionally, we observed significant enrichment of the p53, FoxO, and MAPK signaling pathways, which are likely involved in regulating the DNA damage response and cisplatin resistance (Fig. 7C).

To validate our RNA-seq data, we verified the expression of several genes that we already analyzed in this study. Since the activation of mTORC1 and DNA damage response pathways largely relies on phosphorylation events, we chose to test genes whose total protein products, rather than their phosphorylation states, were deregulated. GATOR1 components themselves, TFEB, and p73 emerged as the best candidates for this analysis. The expression of GATOR1 components was generally lower in the corresponding knockout groups compared to the WT groups under both cisplatin-treated and untreated conditions, confirming the reliability of our knockout experiments and RNA-sequencing results (Fig. S3). At the same time, the amount of DEPDC5 mRNA did not change significantly in *NPRL2*^−/−^ cells (Fig. S3), in contrast to a strong downregulation of the corresponding protein, especially in cells treated with cisplatin (Fig. 1G), suggesting that such a decrease in expression is related to translation and/or protein stability or turnover. The amount of TFEB was increased in *NPRL2*^−/−^ cells, with or without treatment, compared to wild-type cells. Interestingly, the amount of both p73 and ATP7A transcripts was downregulated in all the cells after cisplatin treatment, yet the level of the ATP7A protein did not change, indicating that the decrease in ATP7A transcription is likely due to downregulation of p73 rather than TFEB. Therefore, an increase of ATP7A observed in GATOR1 mutants (Fig. 6B) is not due to transcription, but rather to enhanced translation and/or protein stabilization. LRRC8A, a cisplatin influx transporter, exhibited downregulated mRNA expression in *DEPDC5*^−/−^ cells (Fig. S3), regardless of treatment, likely contributing to the reduced LRRC8A protein levels in these cells (Fig. 6B). Interestingly, in *NPRL3*^−/−^ cells, cisplatin treatment significantly upregulated LRRC8A mRNA expression; however, this increase did not translate to protein expression, as LRRC8A protein levels were diminished. The discrepancy between LRRC8A mRNA upregulation and reduced protein expression warrants further investigation to understand the underlying mechanisms.

In all GATOR1 knockout groups (both with and without cisplatin treatment), relative to the wild-type (WT) group, two genes (*AMOT* and *SERPINB7*) were significantly upregulated, while 13 genes (*FBLN2*, *LYPD1*, *PTGFR*, *SVEP1*, *CD200*, *PTN*, *COL1A1*, *SPOCK1*, *TMSB15A*, *NUBT11*, *SESN3*, *BGN*, and *EPGN*) were significantly downregulated (Fig. 7D). High expression of serine protease inhibitor 7 (SERPINB7) is associated with unfavorable overall survival of patients with cervical cancer [39], as well as patients with pancreatic cancer [40] and NSCLC patients receiving a standard care, including chemotherapy [41]. The role of angiomotin (AMOT) in cancer is currently quite controversial and might depend on the cancer type, because its function both as an oncogene and tumor suppressor was reported [42, 43].

Among downregulated genes, some have tumor suppressive activities, e.g., FBLN2 [44], LYPD1 [45], SVEP1 [46]. For example, FBLN2 is a tumor suppressor gene in NSCLC that inhibits tumor cell survival, proliferation, and invasion through multiple mechanisms, including the suppression of the MAPK/ERK and PI3K/AKT/mTOR signaling pathways [44]. Surprisingly, SESN3 and COL1A1, which are downregulated in GATOR1 deletions (Fig. S4), have pro-tumorigenic activity and their overexpression in cancer is associated with cisplatin resistance [47, 48].

Nevertheless, many genes were downregulated in *NPRL2*^−/−^ cells, known to be related to poor prognosis in various carcinomas and resistance to cisplatin (Fig. 7E). Some of these genes are involved in metabolic functions. For example, in *NPRL2*^−/−^ cells, *PSAT1*, *ASNS*, *GARS*, and *G6PD* were among the upregulated genes with the highest *p* value. PSAT1 [49], ASNS [50], GARS [51], and are implicated in the synthesis of amino acids, while G6PD [52] is important for the synthesis of glutathione, ribose 5’-phosphate, NADPH. At the same time, several genes downregulated in *NPRL2*^−/−^ cells are also known to be downregulated in lung cancer (*FBLN2* [44], *LBH* [53], *AMPH-1* [54]), glioblastoma (*ELAVL2* [55]), and liver cancer (*IGFBP4* [56]). Thus, the deletion of NPRL2 changes the expression of other genes involved in cisplatin resistance. The same tendency is also observed in *DEPDC5*^−/−^ and *NPRL3*^−/−^ cells (Fig. S5). However, a low overlap between differentially expressed genes in GATOR1 deletions (Fig. 7D), suggests that each of GATOR1 components may have a specialized function, different from a role in amino acids responding.

Finally, we compared the transcriptomic profile of BEAS-2B cells bearing GATOR1 deletions with RNA-seq data obtained for A549res and A549par cells, because we used the same cells as in the studies by Sarin et al. [23, 57]. A data set GSE108214 initially produced by Sarin et al. [57] and exploited by other groups [4, 58, 59], annotated many differentially expressed genes within twelve statistically significant GO terms. Our analysis revealed that the DEGs potentially responsible for intrinsic cisplatin resistance (observed in the context of GATOR1 deletion) differ markedly from those associated with acquired cisplatin resistance in A549res cells, although some overlapping genes were identified (Fig. S6, Table S3). Among the overlapping upregulating genes are *G6PD* and *KLF4* (Table S3), known to be related to cisplatin resistance and upregulated in lung and other cancers [60–62]. These findings provide novel insights into the similarities and differences between cells with acquired cisplatin resistance and those with GATOR1 deletion-induced intrinsic resistance cells, opening new directions for future studies.

Collectively, a first RNA-seq dataset of cells with GATOR1 deletions reveals a large number of deregulated genes belonging to the AKT–mTORC1 pathway, metabolism, transcription, and DNA damage response. Changes in GATOR1 expression induce deregulation of genes related to cisplatin resistance and cancer progression.

### NSCLC patients with cisplatin resistance exhibit downregulation of GATOR1 expression

**Fig. 8:**
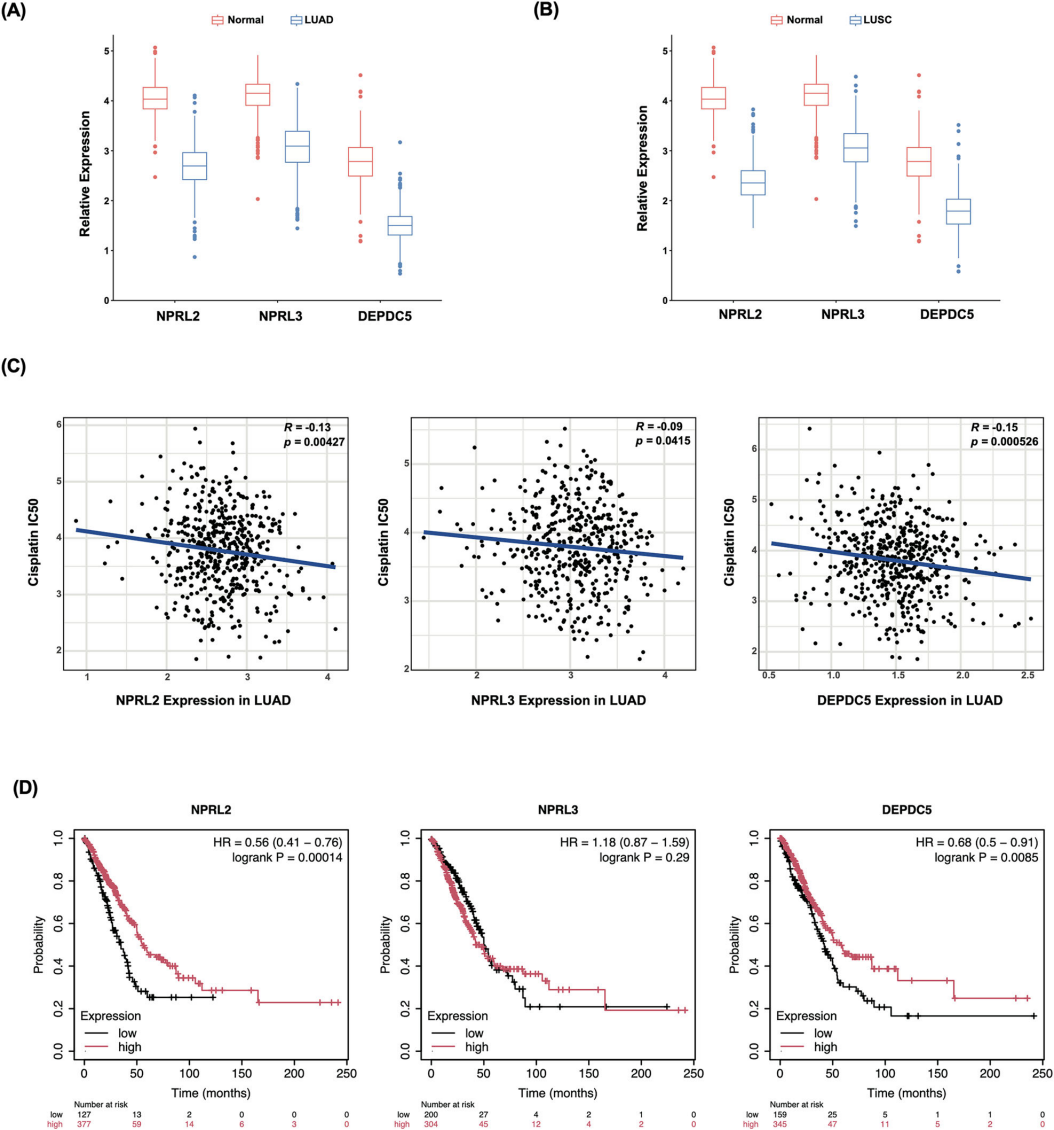
Bioinformatic analysis of GATOR1 expression in different types of NSCLC and its correlation with cisplatin resistance. **A** and **B** Expression pattern of GATOR1 genes in lung adenocarcinoma (LUAD) and lung squamous cell carcinoma (LUSC) compared to normal tissue. **C** Plot charts demonstrating the correlation between the expression of indicated GATOR1 components and cisplatin sensitivity in LUAD. **D** Kaplan–Meier plots showing the correlation between expression of GATOR1 genes and the survival of patients with LUAD. Spearman’s correlation coefficient (*R*) and *p*-value (*p*) are indicated for each case.

The downregulation of NPRL2 and its correlation with cisplatin resistance in lung cancer was initially reported almost two decades ago [20, 63], before it was discovered that NPRL2 belongs to the GATOR1 complex [18]. Whether other components of GATOR1 are deregulated in lung cancer was not addressed until now. Therefore, we analyzed the expression of all three GATOR1 members using publicly available RNA-seq datasets of patients with lung adenocarcinoma (LUAD) and lung squamous cell carcinoma (LUSC), the most common subtypes of NSCLC (Figs. 8, S7). We found that not only NPLR2, but also NPRL3 and DEPDC5 are downregulated in these types of lung cancer (Fig. 8A, B). Moreover, this downregulation was correlated with resistance to cisplatin (Figs. 8C, D, S7) and the lower survival of cancer patients (Figs. 8D, S7).

## Discussion

Despite many advances in cancer treatment, cisplatin resistance remains a major barrier to successful chemotherapy. In this study, we investigated the role of GATOR1 members in mediating cisplatin resistance. We found that deletion of each component of the GATOR1 complex in bronchial epithelial BEAS-2B cells and HEK293T cells increased cellular resistance to the drug. Re-expression of the GATOR1 members in these deleted cells restored their sensitivity to cisplatin.

We also noticed that deletion of any of the GATOR1 subunits reduced protein levels of the other two subunits. However, overexpression of any single GATOR1 subunit did not alter the expression of the other two components in wild-type cells, and, importantly, was also unable to restore the expression of the other two components in the respective deletion cell lines. Therefore, decreased expression of GATOR1 components upon deletion of one subunit reflects destabilization of the complex as a whole, rather than direct regulation at the transcriptional level. Our transcriptomic data further support this, showing that deletion of one GATOR1 component does not affect the mRNA levels of the other two subunits (Fig. S3). Consistently, data from *Drosophila* indicate that knockdown of nprl2 decreases Nprl3 expression in S2 cells or in the ovaries of nprl2 mutants [64]. Similarly, siRNA- or shRNA-mediated downregulation of NPRL2 in HepG2 cells significantly reduces the protein levels of NPRL2 and the other two GATOR1 components, NPRL3 and DEPDC5 [65]. Here, we extend these findings by showing that not only loss of NPRL2, but also downregulation of each GATOR1 subunit individually, leads to decreased expression of the other components.

However, we found that re-expression of the deleted GATOR1 component in knockout cells restored the cisplatin sensitivity phenotype, not only in BEAS-2B cells with GATOR1 deletion. Indeed, overexpression of GATOR1 subunits in various lung cancer cell lines also makes them more sensitive to cisplatin (Fig. 2D). This apparent contrast highlights that while reintroduction of the missing subunit does not re-stabilize the complex partners at the protein level, it can nonetheless restore a subset of functional outputs that are directly mediated by that component. In other words, cisplatin sensitivity may rely on a specific activity of the reintroduced subunit (e.g., interaction with a DNA damage response effector or a signaling node), which does not require full reassembly of the entire GATOR1 complex.

Mechanistically, the deletion of GATOR1 members upregulated the expression of ATP7A, a cisplatin efflux transporter, while diminishing the expression of LRRC8A, a cisplatin influx transporter. The combination of these events leads to impaired accumulation of the drug in the cells and decreased formation of cisplatin–DNA adducts, thus lowering DNA damage. On the other hand, GATOR1 mutants demonstrated stronger DNA damage response and higher mTORC1 activity, resulting in less apoptosis. The first RNA-seq assessment of the gene expression in cells with GATOR1 deletions indicated global changes in metabolic pathways and transcription and provided a valuable data set for further studies. Finally, bioinformatic analysis of LUAD and LUSC, the two most common subtypes of NSCLC, revealed downregulation of all three GATOR1 genes, which was correlated with cisplatin resistance.

One of the most plausible explanations for the important role of GATOR1 in cisplatin resistance might be related to its function as an mTORC1 suppressor. Indeed, mTORC1 is constitutively upregulated in many cisplatin-resistant cancer cells, but the exact mechanisms of this upregulation are not clear [11]. Jiang et al. found that in the senescence-like hepatoma cell line HepG2, RAGC and RHEB, two GTPases acting upstream of mTORC1, but downstream of GATOR1, are required for persistent mTORC1 activity. Knocking down RAGC or RHEB increased cisplatin sensitivity of senescence-like HepG2 cells, which were more resistant to the drug than their proliferating counterparts [66]. In addition, translationally controlled tumor protein (TCTP) can also stimulate mTORC1 by positively regulating RHEB [67]. Our findings regarding the role of GATOR1 add a new element to the explanation of mTORC1 upregulation in cisplatin-resistant cells.

One of the important results that were uncovered during this study is that GATOR1 may influence cisplatin sensitivity not only through its effects on mTORC1 but also via mTORC1-independent functions. In fact, when mTORC1 activity was suppressed by Torin 1 or RapaLink-1, GATOR1 deletion mutants remained slightly more resistant to the drug than wild-type cells (Fig. 3B).

In this study, we report the first RNA-seq analysis of cells with GATOR1 mutations treated or not with cisplatin. This analysis revealed a large number of differentially regulated genes, opening new perspectives in the study of GATOR1’s involvement in tumorigenesis and drug resistance. So far, the only attempt to evaluate differentially expressed genes in cells with GATOR1 deletions was made almost a decade ago by performing microarray analysis of breast cancer cell lines expressing or not expressing NPRL2, detecting a high proportion of genes involved in cell cycle regulation and DNA damage response [68].

In NPRL2-deleted cells (both with and without cisplatin treatment), *PSAT1*, *ASNS*, *G6PD*, *DPYD*, and *SEPT*9 were among the top-upregulated genes (Fig. 6D). Upregulation of PSAT1 in cervical, ovarian, and colorectal cancers activates the PI3K/AKT signaling pathway, thereby improving cellular redox balance and enhancing the repair of cisplatin-induced DNA damage [49, 69, 70]. High ASNS expression has been linked to cisplatin resistance and poor prognosis in gastric cancer patients [71]. G6PD enhances NADPH production, which is associated with poor tumor prognosis [52]. Both DPYD [72] and CircSEPT9 [73], a circular RNA formed by back-splicing of SEPT9 exons, have been implicated in cisplatin resistance. The significant downregulation of COL1A1, COL1A2, and SMOC1 suggests impaired extracellular matrix integrity in *NPRL2*^−/−^ cells [74] (Fig. 7E). Among the top downregulated DEGs in *NPRL3*^−/−^ and *DEPDC5*^−/−^ cells (Fig. S5), IGFBP3 [75], and WNT5A [76] were found to be associated with cisplatin resistance. COL1A1 was also downregulated in these cells (Fig. S4).

The downregulation of collagen type 1 alpha 1 (COL1A1) in GATOR1-deleted cells represents a somewhat paradoxical behavior of certain DEGs that we uncovered in this study. COL1A1 is typically overexpressed in numerous cancers, where it is often associated with aggressive disease and poor prognosis. mTORC1 is implicated in COL1A1 expression via the 4E-BP1 signaling axis, which is essential for transforming growth factor-beta 1 (TGF-β1)-induced collagen synthesis [77, 78]. In lung fibroblasts, mTOR suppression with Torin 1 inhibits COL1A1 expression both at mRNA and protein levels [75], which is in agreement with our data (Fig. S4). Additionally, cisplatin treatment also completely abolished COL1A1 expression. Interestingly, in BEAS-2B cells, we observed that both mTORC1 suppression by Torin1 and its persistent hyperactivation due to GATOR1 deletion led to the suppression of COL1A1, which probably means that GATOR1 action on COL1A1 transcription is mTORC1-independent. One of the few examples of COL1A1 downregulation in cancer was reported for hepatocellular carcinoma, where COL1A1 expression was significantly decreased in tumor tissue compared to adjacent normal tissue, with promoter hypermethylation identified as the silencing mechanism [79]. Whether the same mechanism is implicated in GATOR1-deleted cells or whether other transcriptional factors and modulators of COL1A1 are involved remains to be determined.

Previously, several microarray analyses have been reported for A549 cells treated with cisplatin. Galluzi et al. only studied sensitive A549 cells without comparing them with resistant cells, validating potential genes important for resistance in yeast *S. cerevisiae* [80]. Yang et al. performed microarray expression profiling in both sensitive and resistant A549 cells and revealed that, in addition to a large number of differentially expressed mRNAs, there were almost as many long non-coding RNAs changing their expression profiles [81]. Finally, Sarin et al. explored the transcriptome of sensitive and resistant cells in different treatment conditions [57], but did not address the expression of non-coding RNAs, which have recently gained a lot of attention due to their ability to inhibit autophagy and increase the sensitivity of cancer cells to cisplatin [11]. BEAS-2B cells with GATOR1 deletions demonstrate several deregulated genes in common with the above studies (Table S3). In addition, the expression of DEPDC5 is downregulated in A549 (Fig. 2C).

One must be cautious when interpreting transcriptomic data for individual genes, because changes in mRNA expression do not always correlate with the production of proteins, which can reflect the problems of translation or protein degradation. For example, concurrent analysis of mRNA expression and protein profiling in resistant and sensitive ovarian carcinoma cells revealed a discrepancy in mRNA and protein expression in 55% of the proteome [82]. Similar observations have been made during the analysis of A549 resistant and sensitive cells [57]. The RNA-seq dataset for GATOR1-deleted cells also exhibits this trend (see examples of ATP7A and LRRC8A mRNA and protein expressions (Figs. 5, S3)), but it remains a valuable resource for exploring the various mTORC1-related and independent functions of GATOR1 components. This will be particularly important because, despite being the components of the same complex, NPRL2, NPRL3, and DEPDC5 appear to have slightly different functions, as evidenced by a large number of unique DEGs for each mutant (Fig. S2) and their varied responses to DNA damage (Fig. 4C).

Resistance to cisplatin has been initially observed in yeast *S. cerevisiae* for Npr2 and Npr3 deletion mutants [83] (NPRL2 and NPRL3 homologs, respectively), years before GATOR1 components were suggested to be tumor suppressors [18, 20]. Later, many reports confirmed the role of NPRL2 as a tumor suppressor not only in NSCLC, but also in renal, ovarian, colorectal, breast, and other cancers [17]. Overexpression of NPRL2 in NPRL2-deficient and cisplatin-resistant NSCLC cells reactivated cellular response to cisplatin treatment and promoted tumor suppression activity in vitro and in mouse models [20]. NPRL2 overexpression also suppresses tumor growth in colorectal cancer cell lines and xenograft mice, increasing sensitivity to 5-fluorouracil and oxaliplatin [84]. There are fewer articles describing the role of NPRL3 and DEPDC5 in cancers, probably because NPRL2 has a higher cancer-associated mutation frequency among GATOR1 components [85]. However, DEPDC5 inactivating mutations were detected in glioblastoma [18], breast cancer [86], and gastrointestinal stromal tumors [87].

GATOR1 members are also involved in the resistance to other anticancer drugs. For example, colon cancer cells with homozygous deletion of DEPDC5 exhibited resistance to 5-fluorouracil, a first-line chemotherapeutic in colorectal cancer, and to MK2206, a pan-AKT inhibitor [88]. Overexpression of NPRL2 in colon cancer cells increased the sensitivity to a topoisomerase I inhibitor, irinotecan (CPT-11), by activating the DNA damage checkpoints [89]. NPRL2 gene therapy can also sensitize immunotherapy-resistant NSCLC tumors in humanized mouse models [90]. Genomic alternation of all three GATOR1 components is also associated with resistance to PI3Kα inhibitors in primary and metastatic breast cancer [91]. In this case, resistance is explained by the sustained activation of the mTORC1 pathway due to loss-of-function mutations in GATOR1 members.

In summary, our results support a role for all three components of the GATOR1 complex in cisplatin resistance. An improved understanding of cisplatin resistance will better identify therapeutic targets and allow a more accurate prediction of clinical response.

## Supplementary information

Supplementary Figures and Tables legends

Figure S1 - revised

Figure S2

Figure S3

Figure S4

Figure S5

Figure S6

Figure S7

Original uncropped blots

Table S1

Table S2

Table S3

## Data Availability

The RNA-seq raw data have been deposited in the European Nucleotide Archive (ENA) under accession number PRJEB98686. Any additional information required to reanalyze the data reported in this paper is available from the lead contact upon request.
