# Supplementary material for: GATOR1 complex controls cisplatin sensitivity
Source: Cell Death Dis. 2025 Dec 30;17(1):58. doi: 10.1038/s41419-025-08392-4 (PMC12824275; doi:10.1038/s41419-025-08392-4)
Supplement: Supplementary file 1 — Supplementary Figures and Tables legends [file 41419_2025_8392_MOESM1_ESM.docx]

**Supplementary Figures and Tables legends**

**Figure S1 (related to Figure 1).** HEK293T cells with deletions of GATOR1 components are resistant to cisplatin. (A) Cell viability test by MTT assay for each homozygous deletion of the indicated GATOR1 components. Cells were treated with 50 μM cisplatin for 24 h. (B) Western blot analysis of the expression of different GATOR1 components in the indicated depletion cell lines. (C) Cell proliferation assay in A549 cisplatin sensitive and resistant cell lines. Cells were treated with 10 μM cisplatin. See Materials and Methods for details.

**Figure S2 (related to Figure 6).** Bar graphs of GSEA analysis results for the *NPRL2-/-* group compared to the WT group (left) and the same pair of groups treated with cisplatin (right). The x-axis represents the Normalized Enrichment Score, with blue indicating GO pathways and red indicating KEGG pathways.

**Figure S3 (related to Figure 6).** Boxplots illustrating the expression levels (gene counts) of the indicated genes across various BEAS-2B cell groups, including the wild-type (WT, red) and GATOR1 component knockout (green) groups. N2 – *NPRL2-/-*, N3 – *NPRL3-/-*, D5 – *DEPDC5-/*-, CDDP – cisplatin. Horizontal bars above the boxplots denote pairwise statistical comparisons, with significance levels indicated as follows: ns (not significant), * (0.01 ≤ p < 0.05), ** (0.001 ≤ p < 0.01), and *** (p < 0.001).

**Figure S4** **(related to Figure 6).** COL1A1 mRNA and protein expression in BEAS-2B cells with or without GATOR1 deletions. (A) Boxplots illustrating the expression levels (gene counts) of the indicated genes across various BEAS-2B cell groups, including the wild-type (WT, red) and GATOR1 component knockout (green) groups. N2 – *NPRL2-/-*, N3 – *NPRL3-/-*, D5 – *DEPDC5-/-*, CDDP – cisplatin. Horizontal bars above the boxplots denote pairwise statistical comparisons, with significance levels indicated as follows: ns (not significant), * (0.01 ≤ p < 0.05), ** (0.001 ≤ p < 0.01), and *** (p < 0.001). (B) Expression of COL1A1 was tested by western blot in the indicated cells treated or not with cisplatin (50 μM, 24h), Torin 1 (1 μM, 2h) or combination of drugs (treatment with Torin 1 for 2h, then cisplatin addition for 24h). Mw – molecular weight of probed proteins. Representative western blot images from three independent experiments are shown.

**Figure S5** **(related to Figure 6).** Volcano plots display the DEG analysis results for different groups of BEAS-2B cells. The top left panel shows the comparison between the *DEPDC5-/-* group and the WT group without cisplatin treatment, while the top right panel represents the same comparison after cisplatin treatment. Similarly, the bottom left and right panels illustrate the comparisons for the *NPRL3-/-* group versus the WT group under untreated and cisplatin-treated conditions, respectively. The x-axis represents Log2(Fold Change), while the y-axis represents -Log10(Adjusted p-value). The two dashed lines indicate the thresholds for filtering DEGs with Adjusted p-value < 0.01 and |Log2(Fold Change)| > 1. Red points represent up-regulated genes, blue points represent down-regulated genes, and gray points indicate genes with no significant differences. Among the up/down-regulated genes, the top 5 gene symbols with the highest -Log10(Adjusted P-Value) and |Log2(Fold Change)| are marked on the plot.

**Figure S6 (related to Figure 6).** Venn diagrams showing the intersections between DEGs identified from comparisons of A549 cisplatin‐resistant versus cisplatin‐sensitive groups under both cisplatin‐treated and untreated conditions, and DEGs derived from GATOR1 component depletion groups (relative to the WT group) under analogous treatment conditions. Panels A and B correspond to the *DEPDC5-/-* group, Panels C and D to the *NPRL2-/-* group, and Panels E and F to the *NPRL3-/-* group. In each panel, the left diagram represents the up-regulated DEGs, whereas the right diagram displays the down-regulated DEGs. Each circle signifies a DEG set from one comparison, and the overlapping regions denote the common DEGs shared between groups, with the numbers within these intersections indicating the count of shared DEGs.

**Figure S7** **(related to Figure 7).** (A) Bioinformatic analysis of GATOR1 expression in lung squamous cell carcinoma (LUSC) compared to normal tissue. (B) Kaplan-Meier plots showing the correlation between the expression of GATOR1 genes and the survival of patients with LUSC. Spearman’s correlation coefficient (R) and p-value (p) are indicated for each case.

**Table S1.** Comparison of differentially expressed genes and transcription factors in various GATOR1 depleted cells.

**Table S2.** Top 10 up- and down-regulated transcription factors in GATOR1 depletions BEAS-2B cells treated or not with cisplatin.

**Table S3.** Gene symbols contained within the central intersections of Venn diagrams in Fig. S5. Within each column, the gene symbols are arranged from top to bottom in descending order according to their significance in the differential analysis.
