# Supplementary figures and images for: GATOR1 complex controls cisplatin sensitivity
Source: Cell Death Dis. 2025 Dec 30;17(1):58. doi: 10.1038/s41419-025-08392-4 (PMC12824275; doi:10.1038/s41419-025-08392-4)

(A)

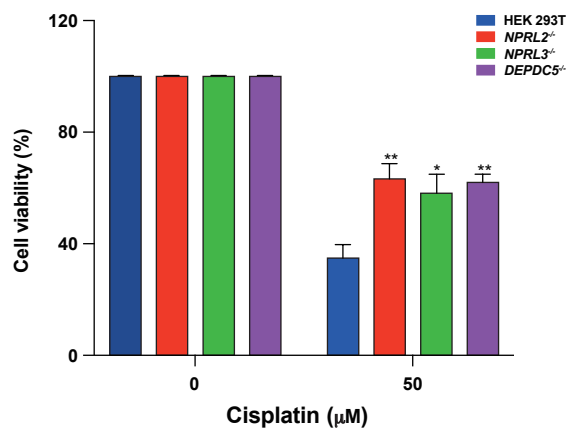

(B)

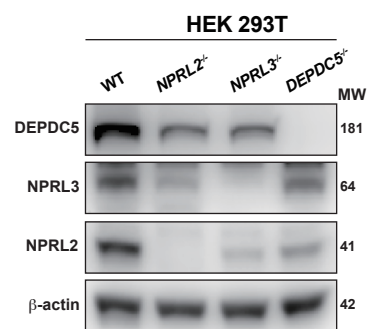

(C)

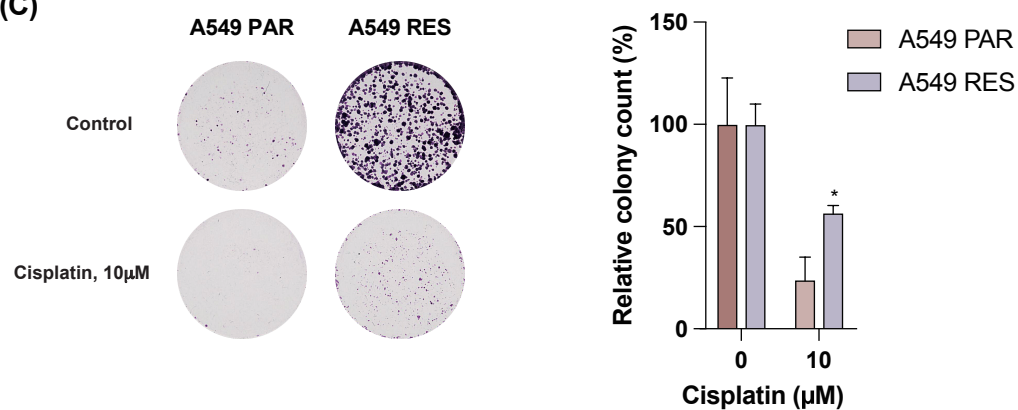

Supplement: Supplementary file 2 — Figure S1 - revised [file 41419_2025_8392_MOESM2_ESM.pdf]

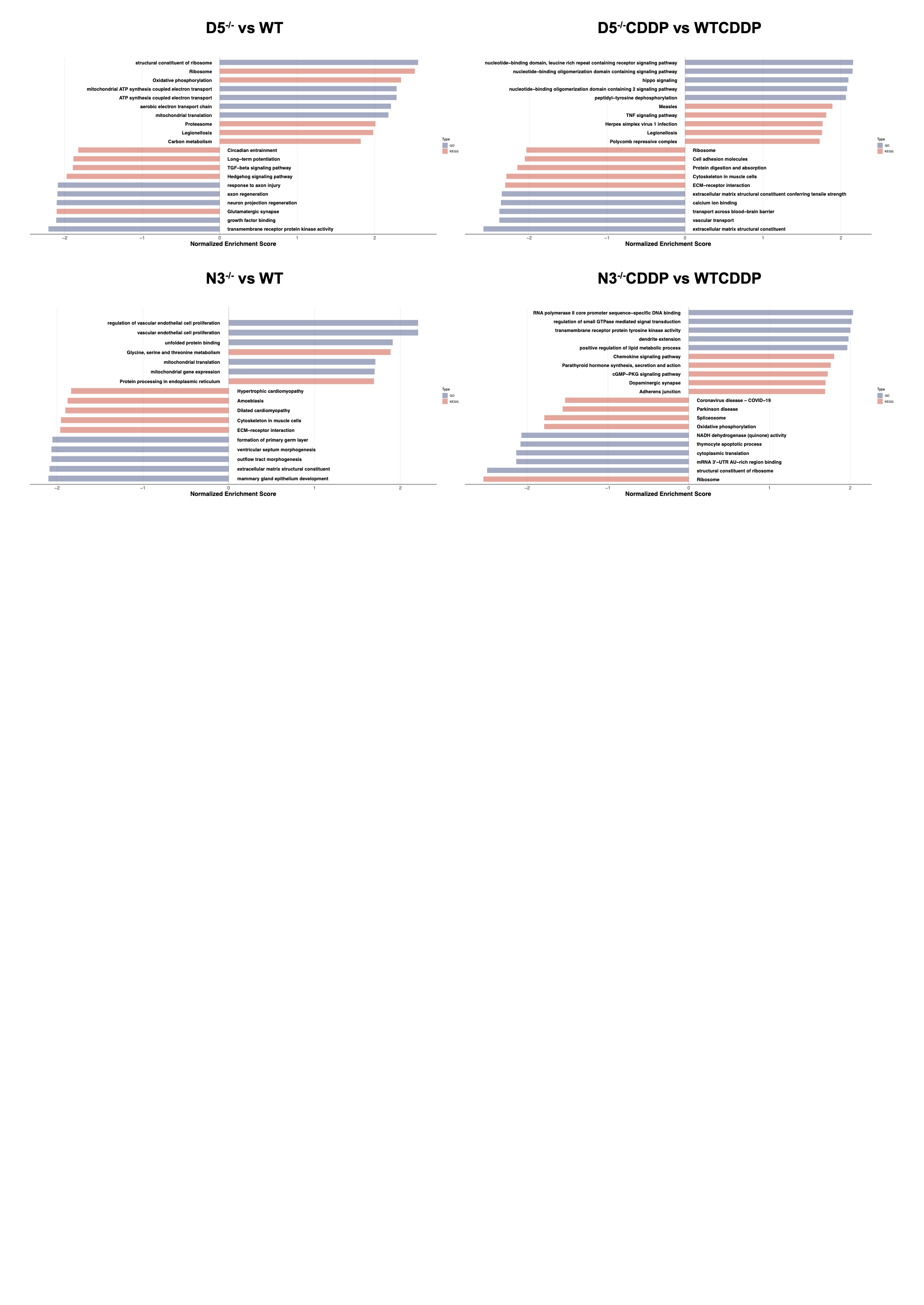

Supplement: Supplementary file 3 — Figure S2 [file 41419_2025_8392_MOESM3_ESM.png]

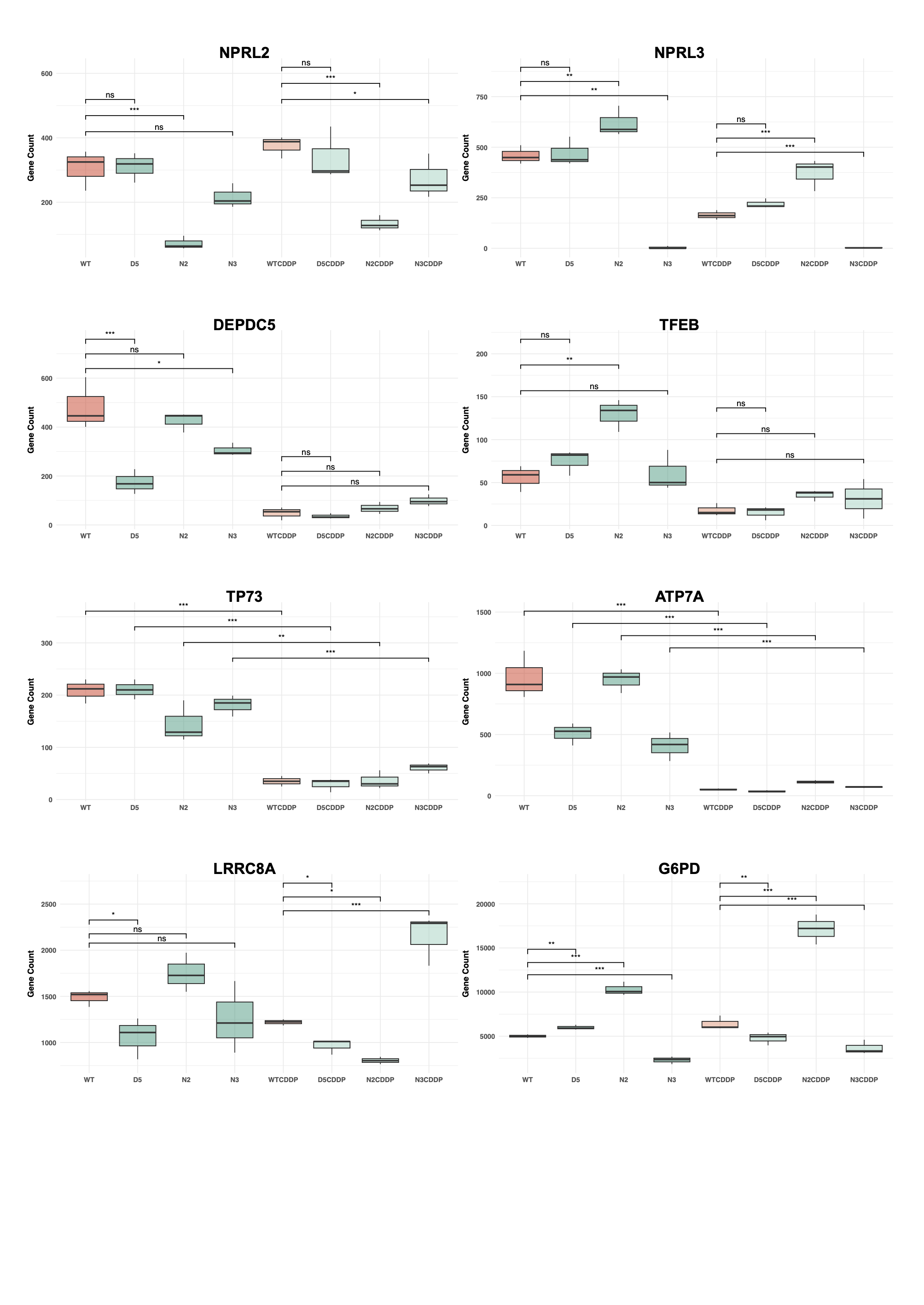

Supplement: Supplementary file 4 — Figure S3 [file 41419_2025_8392_MOESM4_ESM.png]

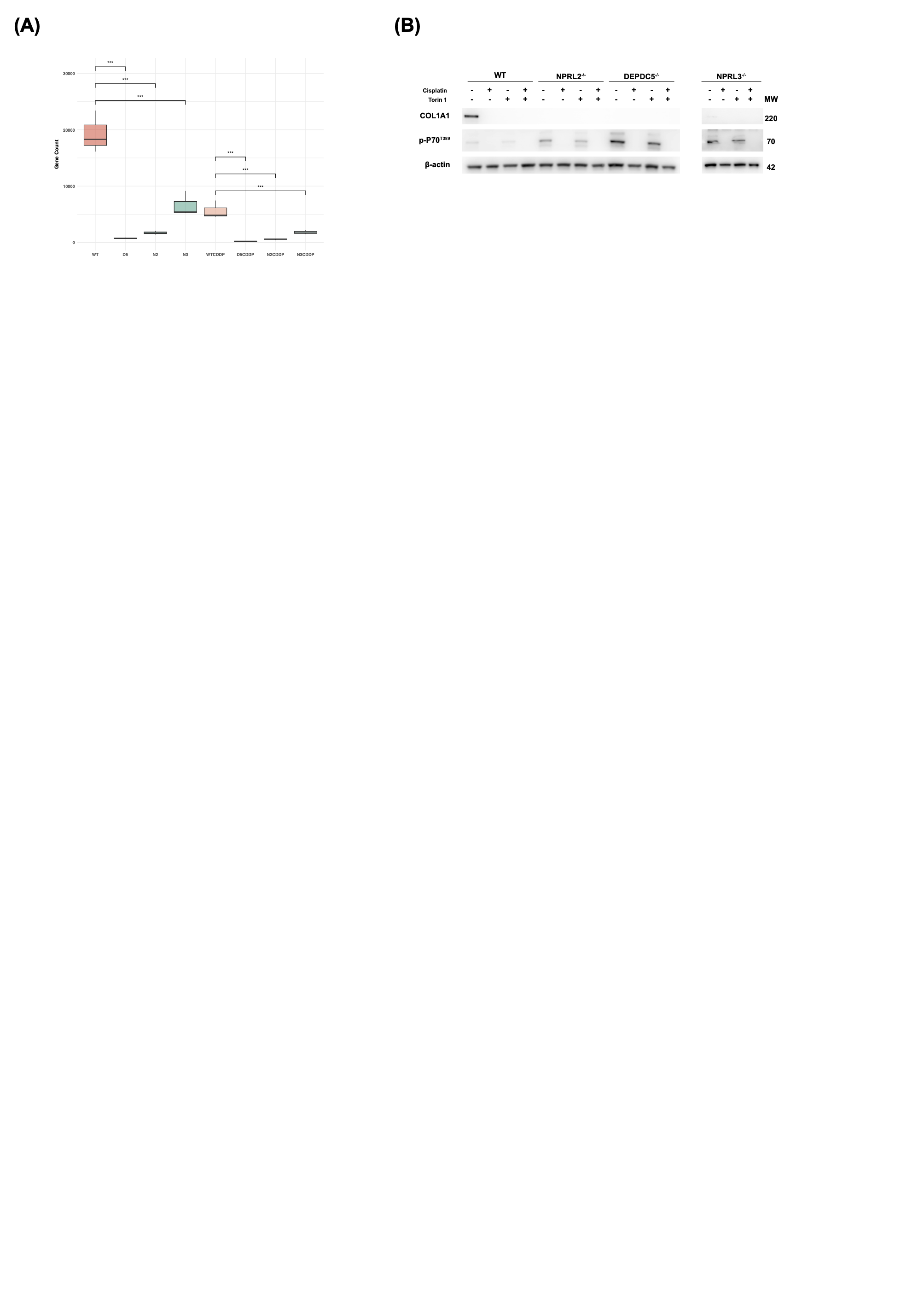

Supplement: Supplementary file 5 — Figure S4 [file 41419_2025_8392_MOESM5_ESM.png]

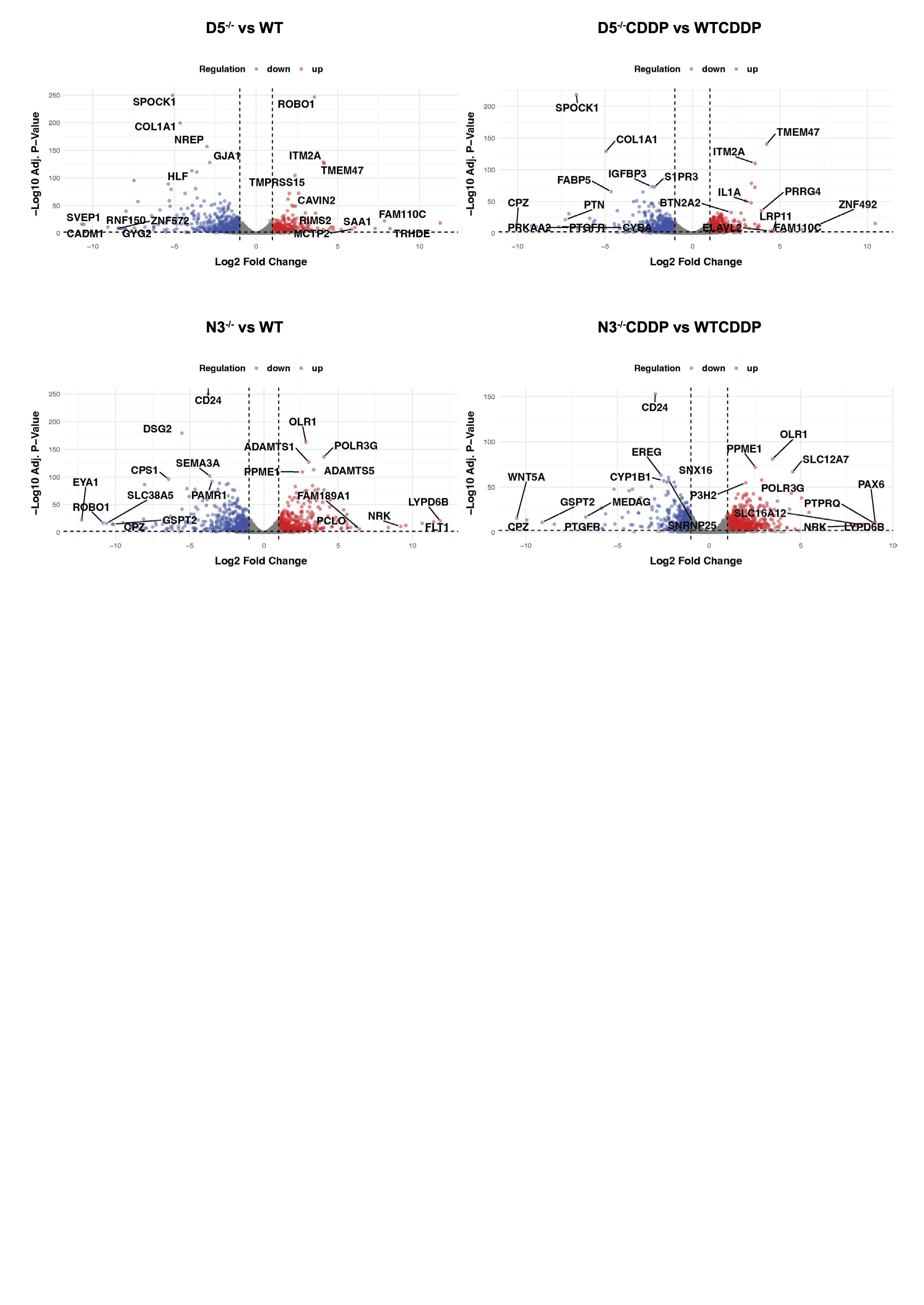

Supplement: Supplementary file 6 — Figure S5 [file 41419_2025_8392_MOESM6_ESM.png]

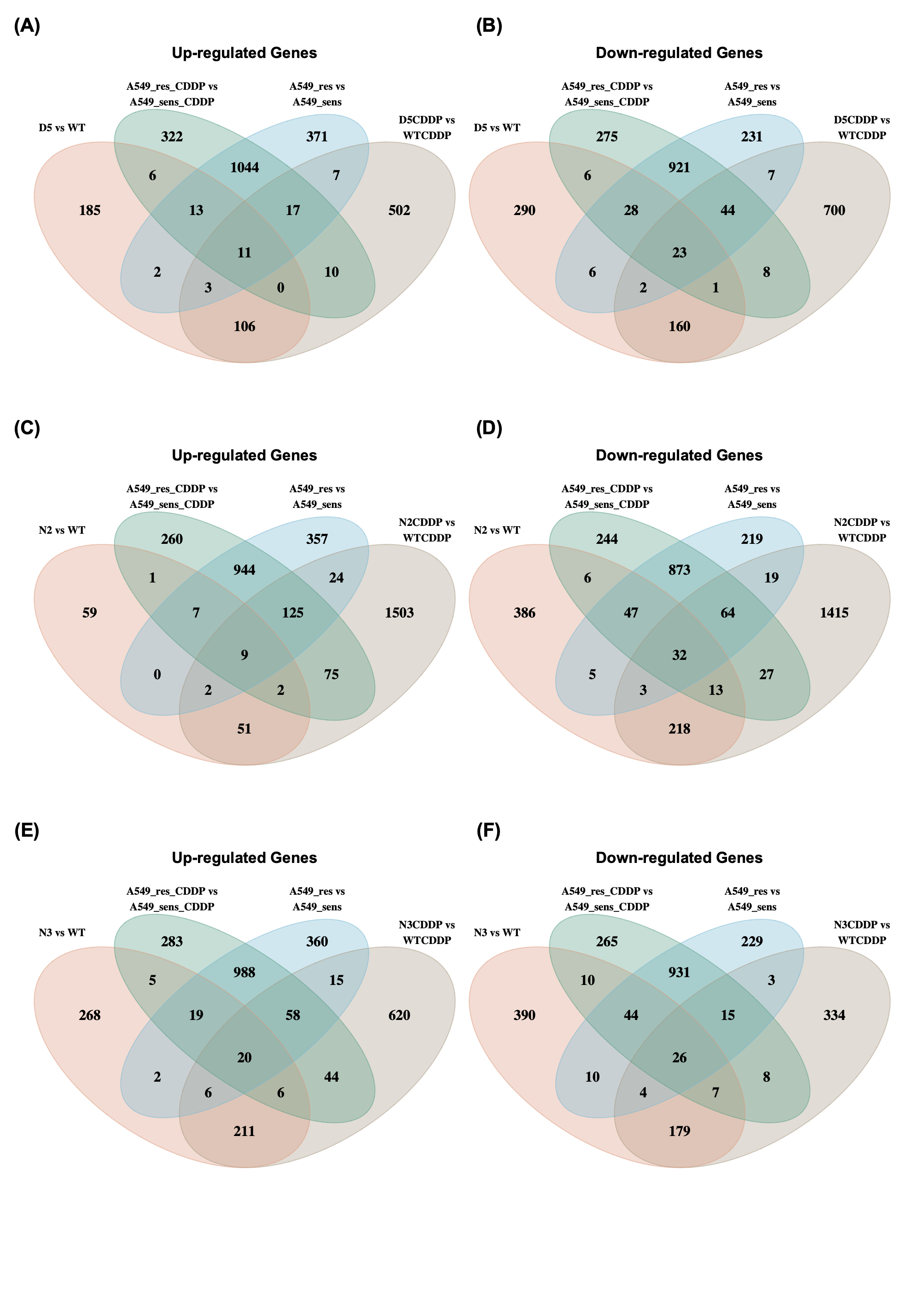

Supplement: Supplementary file 7 — Figure S6 [file 41419_2025_8392_MOESM7_ESM.png]

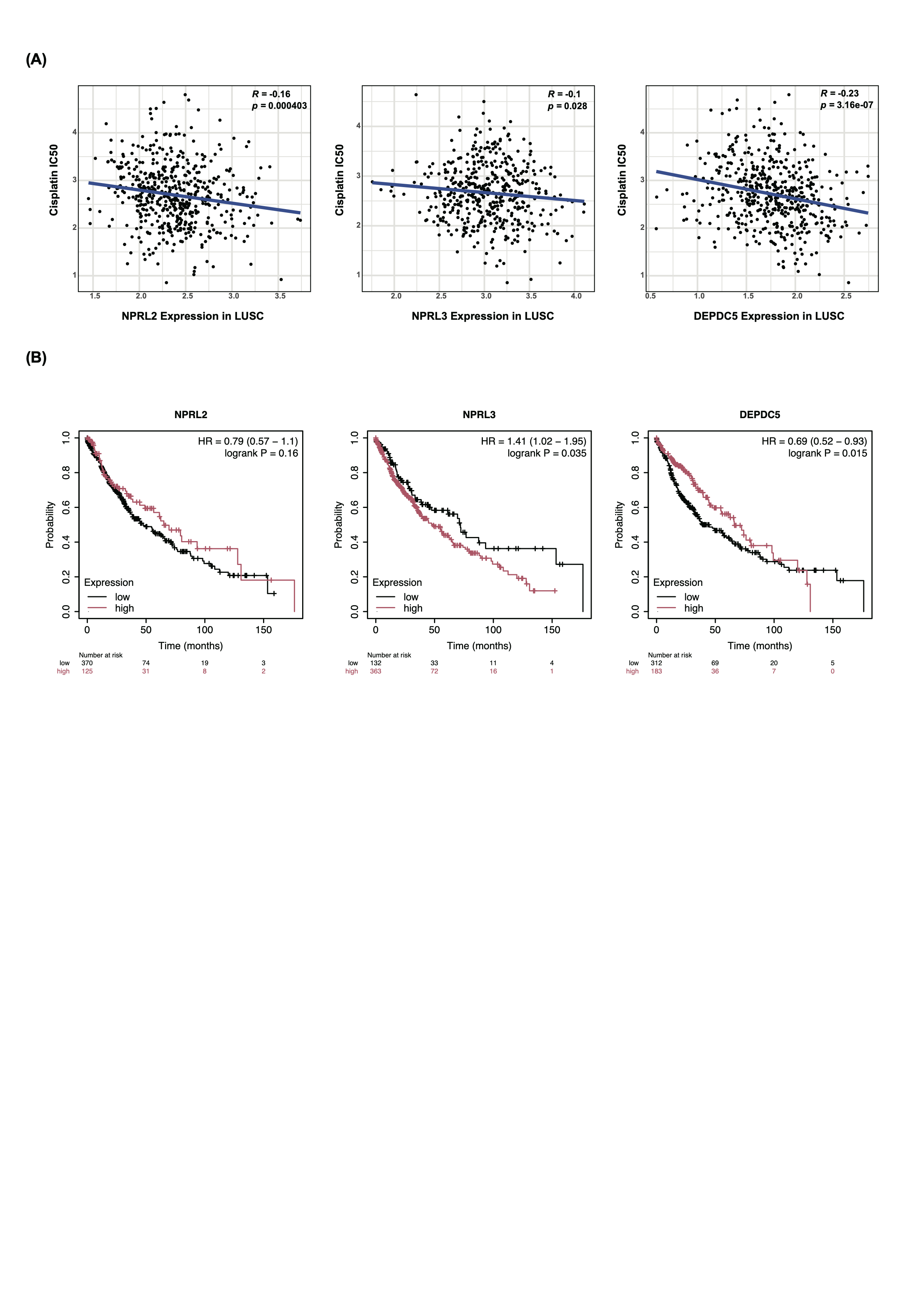

Supplement: Supplementary file 8 — Figure S7 [file 41419_2025_8392_MOESM8_ESM.png]
