# Supplementary material for: GATOR1 complex controls cisplatin sensitivity
Source: Cell Death Dis. 2025 Dec 30;17(1):58. doi: 10.1038/s41419-025-08392-4 (PMC12824275; doi:10.1038/s41419-025-08392-4)

The whole uncropped images of the original blots in Fig1E.

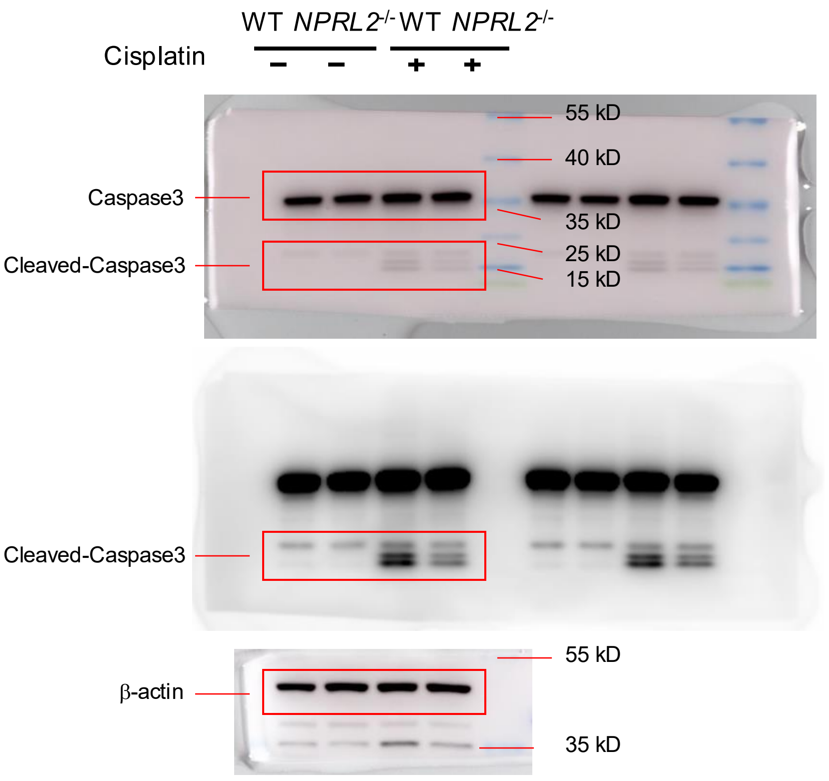

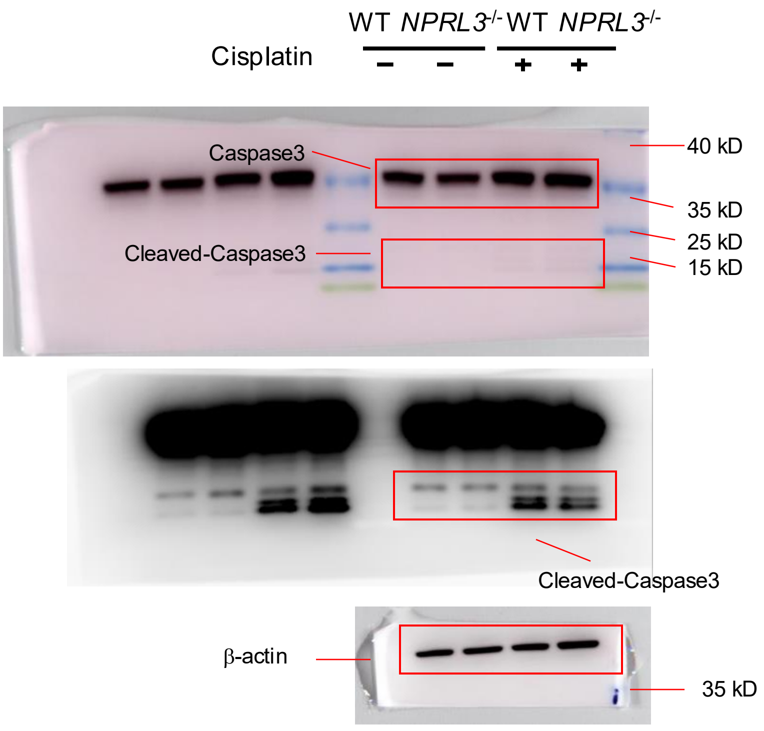

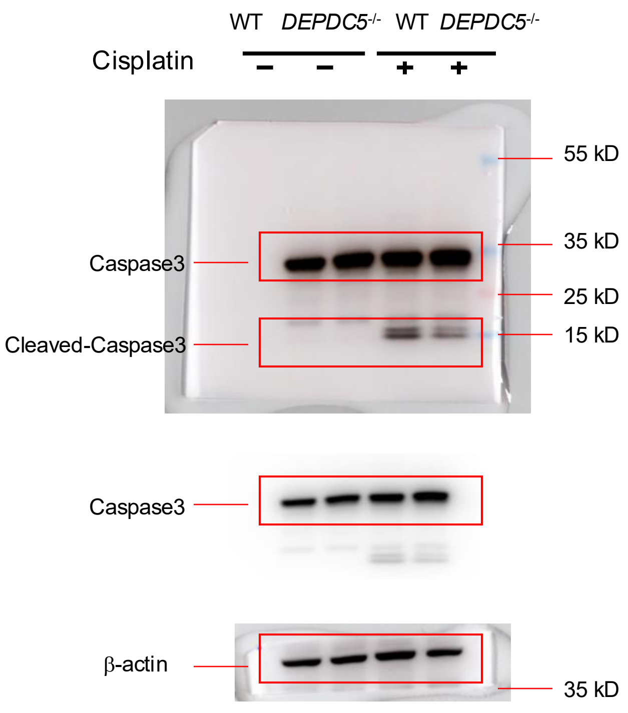

The whole uncropped images of the original blots in Fig1F.

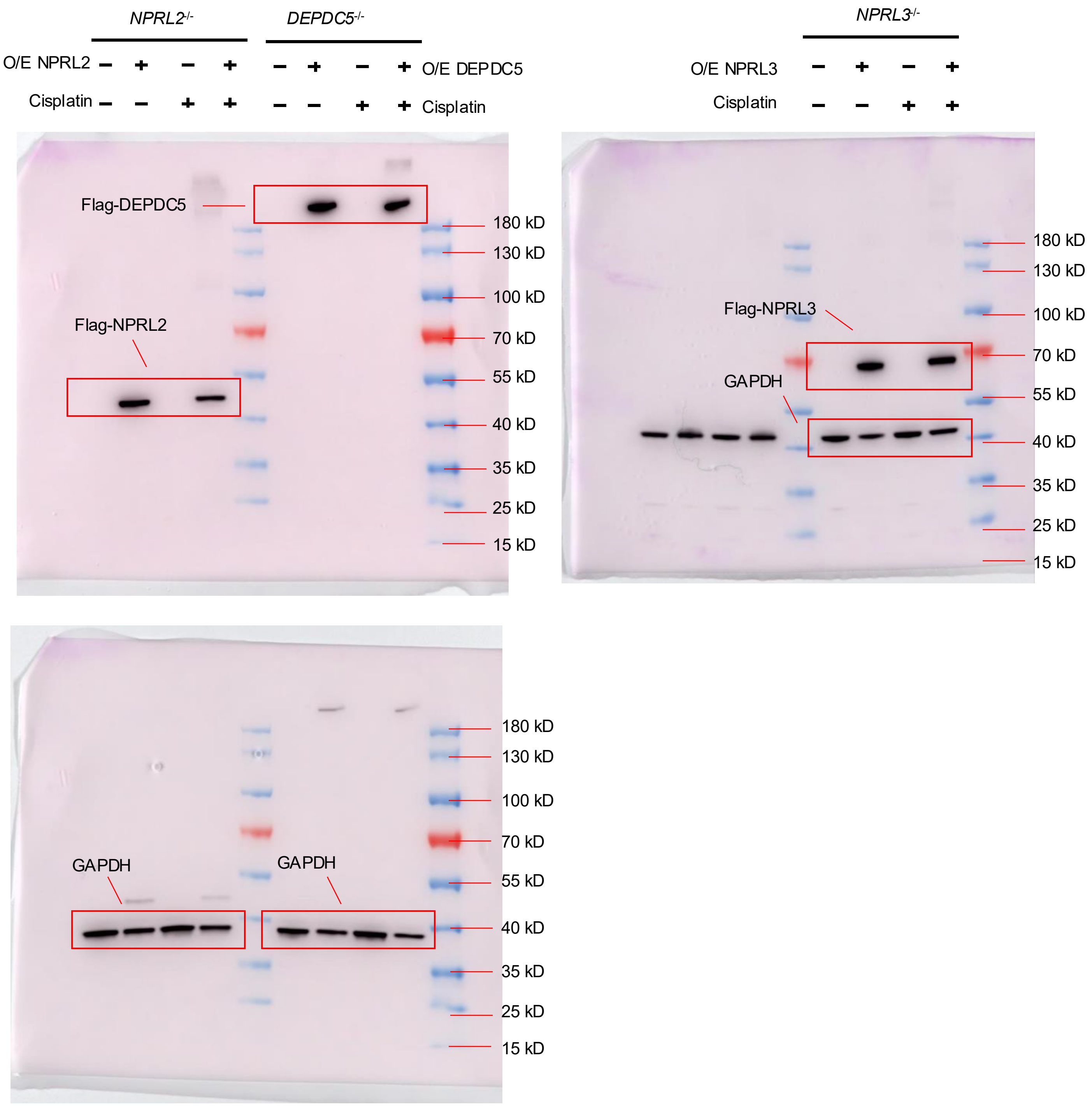

The whole uncropped images of the original blots in Fig1G.

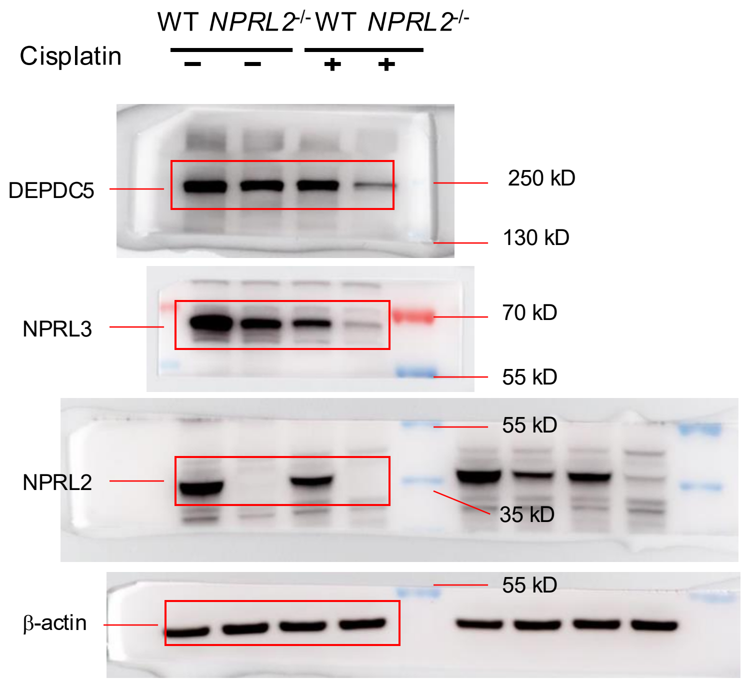

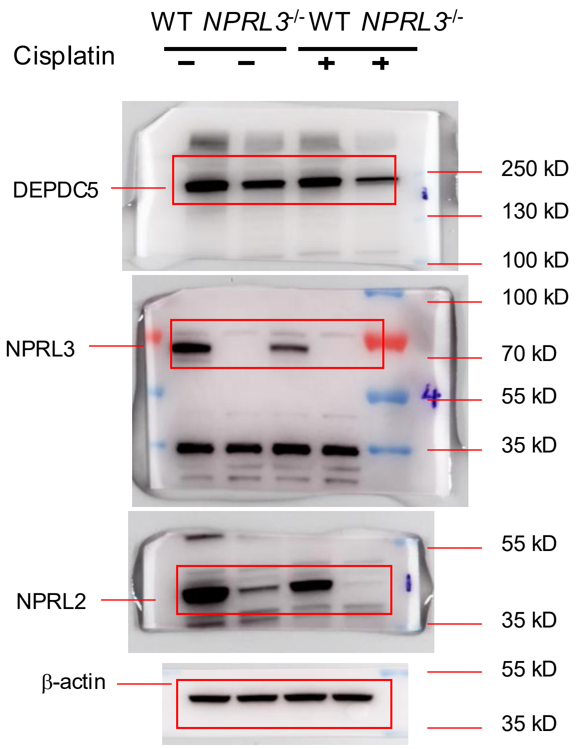

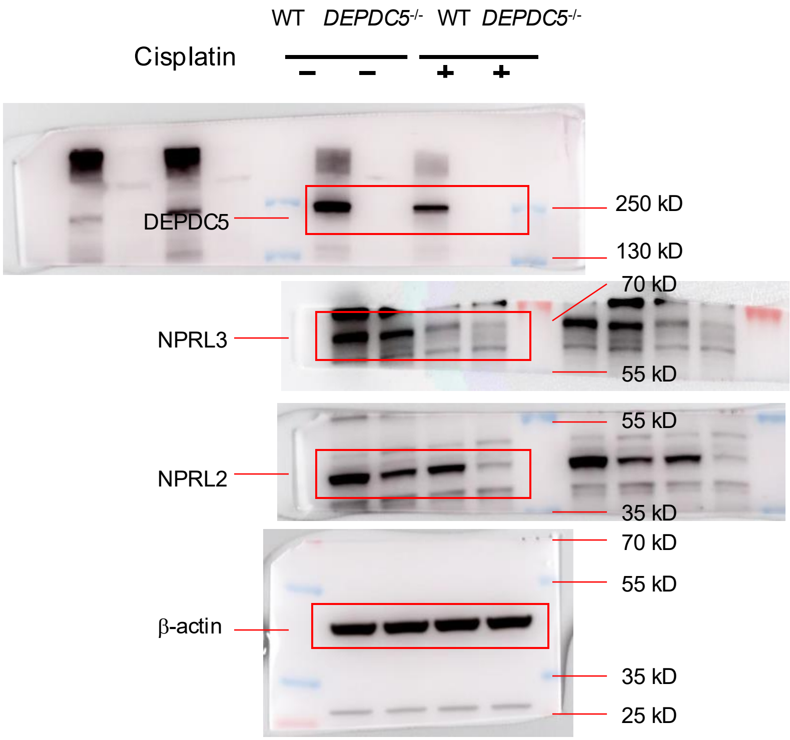

The whole uncropped images of the original blots in Fig1H.

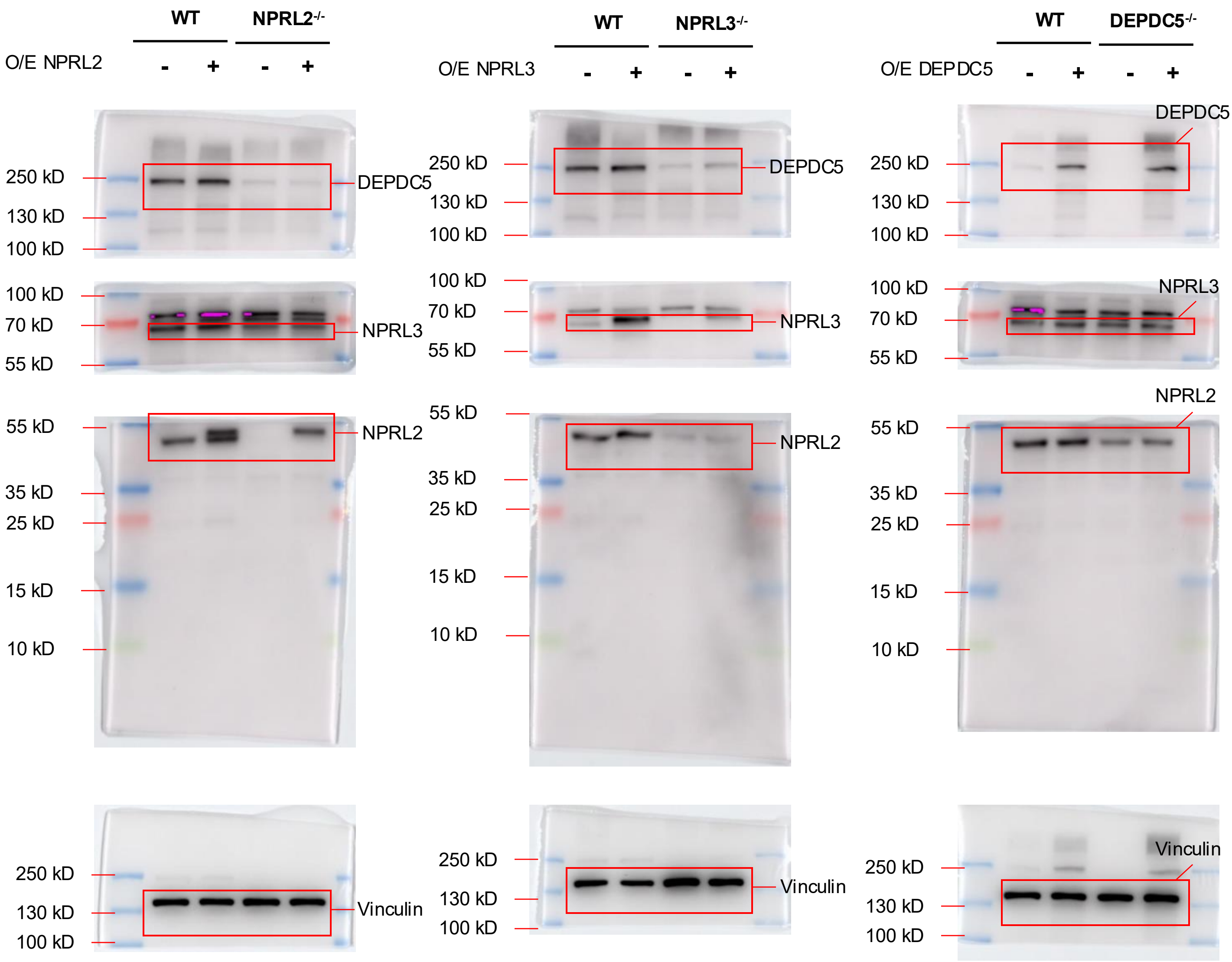

The whole uncropped images of the original blots in Fig2C.

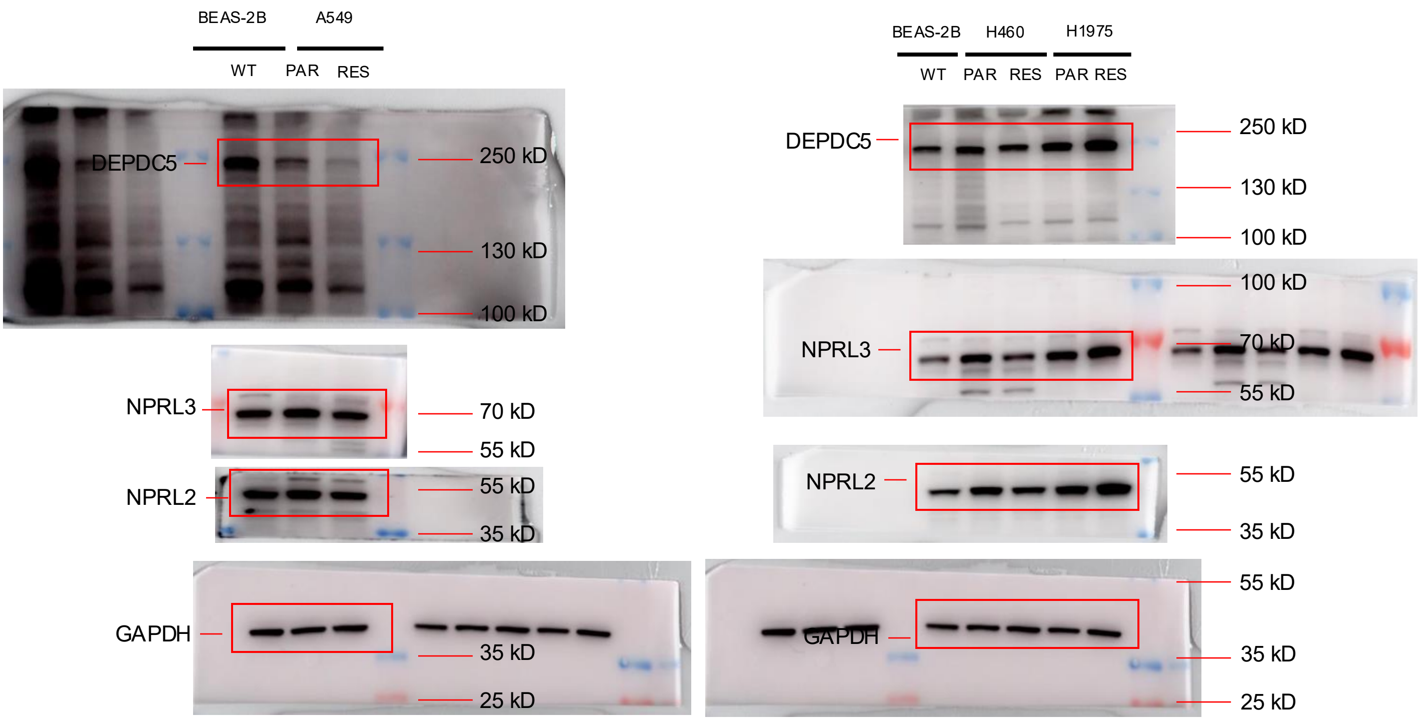

The whole uncropped images of the original blots in Fig2D.

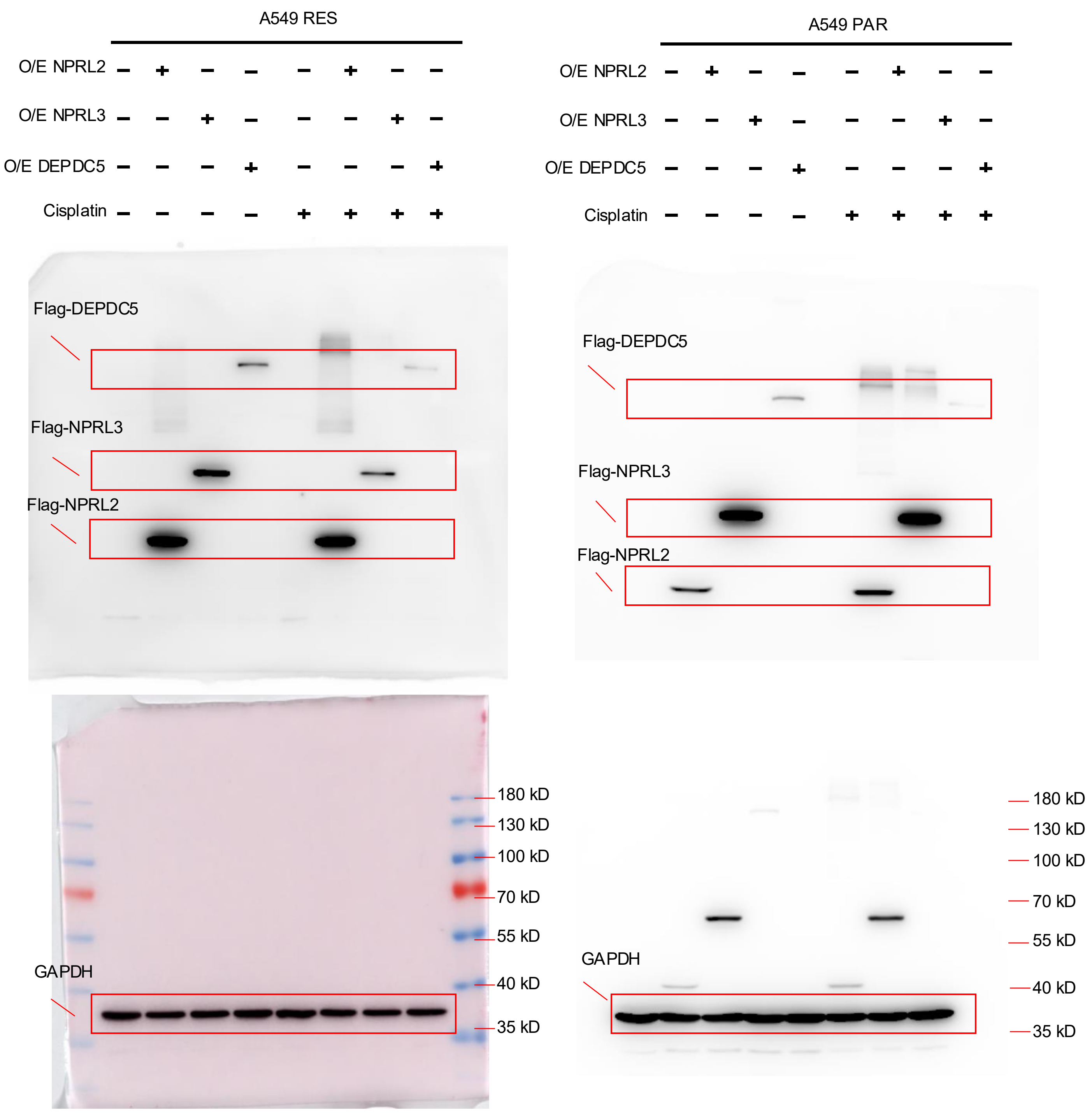

The whole uncropped images of the original blots in Fig2D.

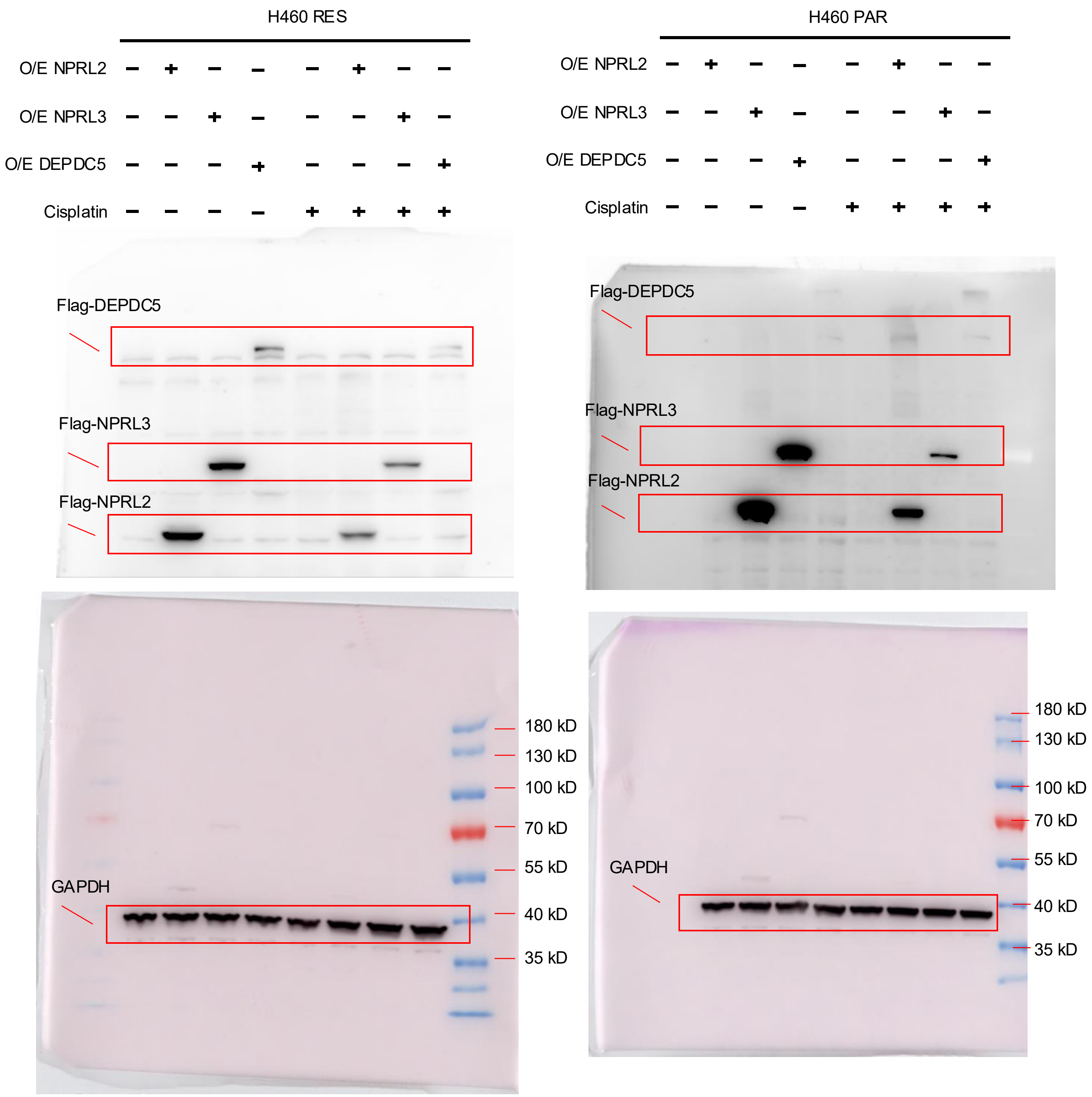

The whole uncropped images of the original blots in Fig2D.

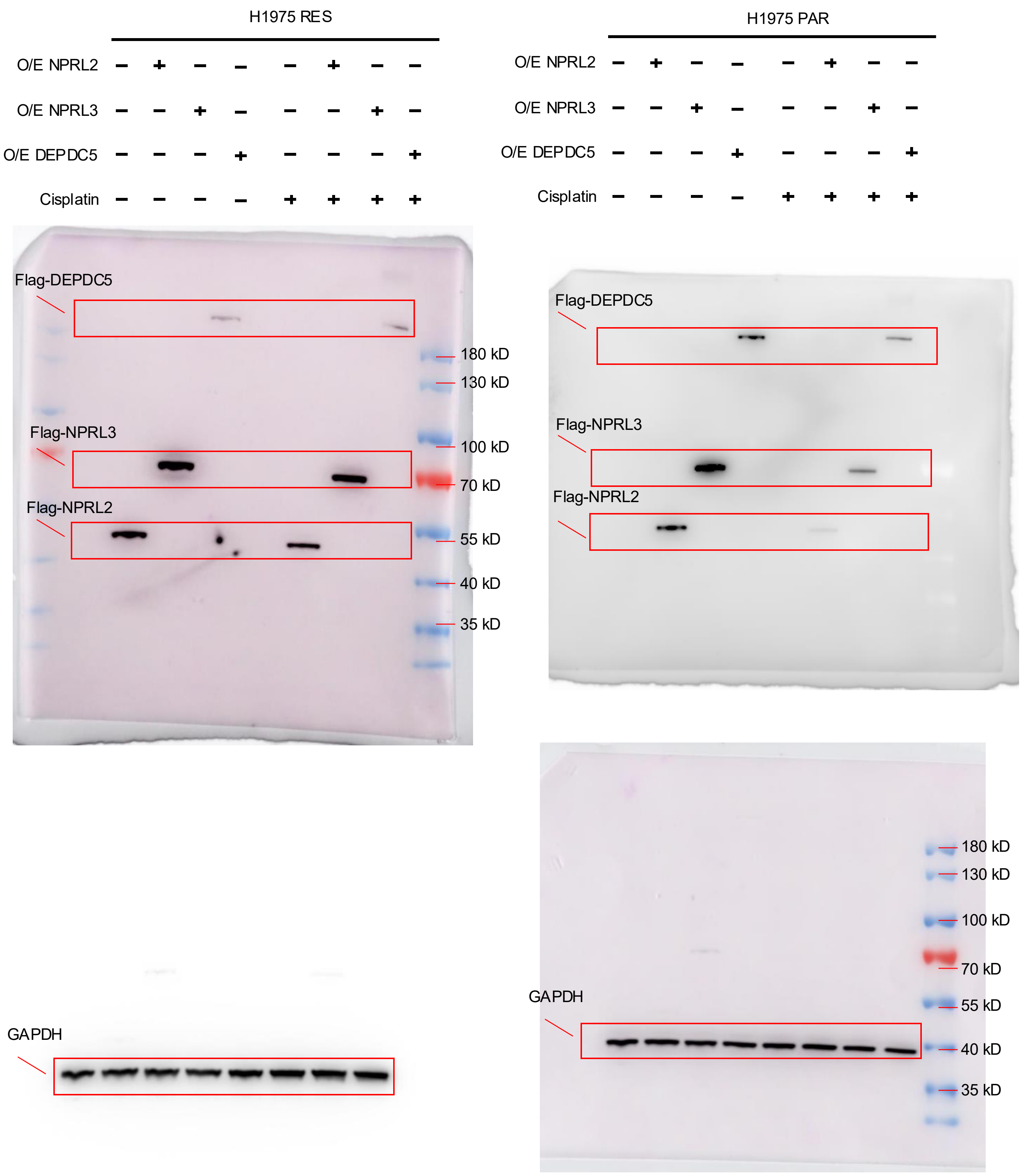

The whole uncropped images of the original blots in Fig3A.

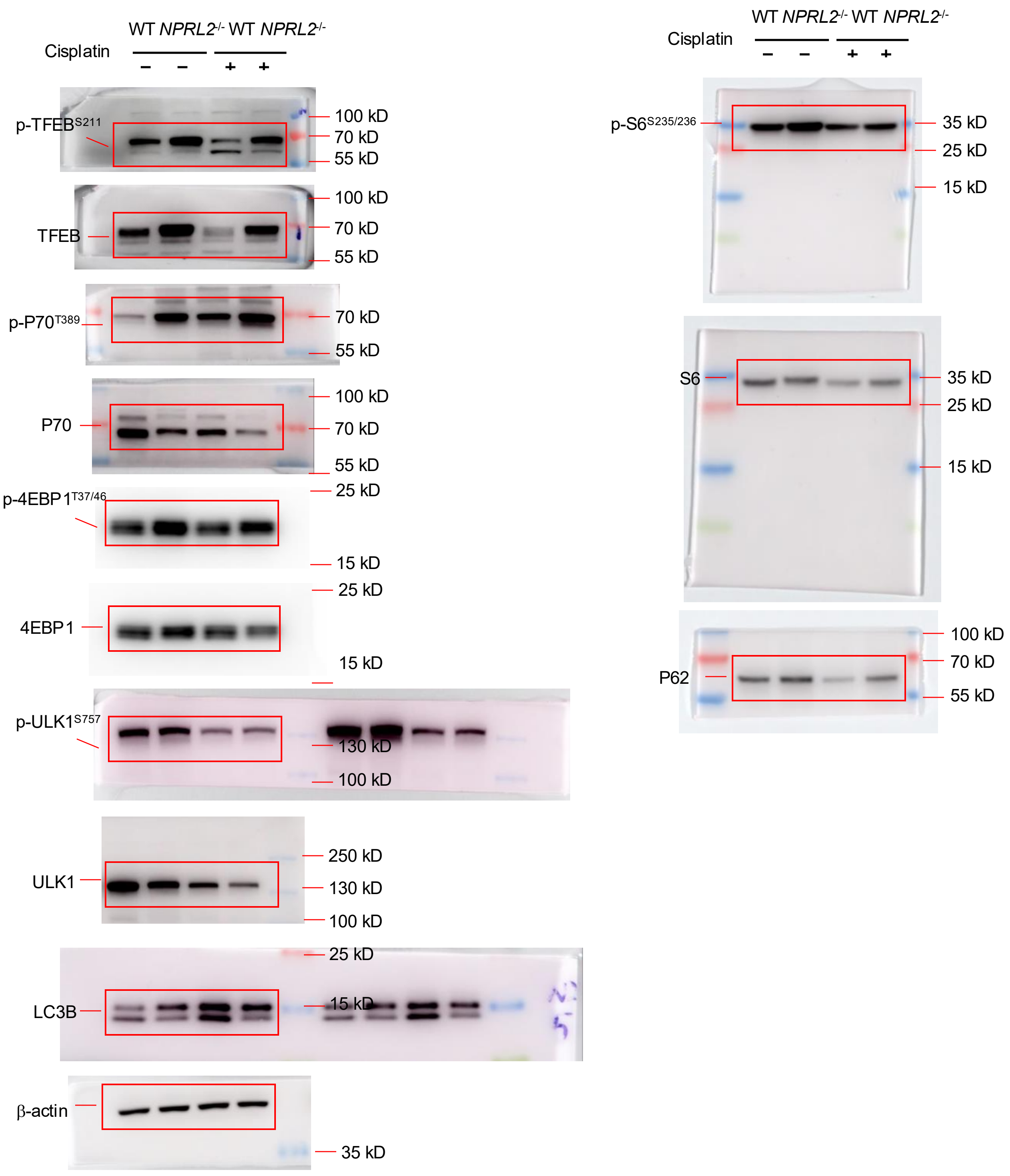

The whole uncropped images of the original blots in Fig3A.

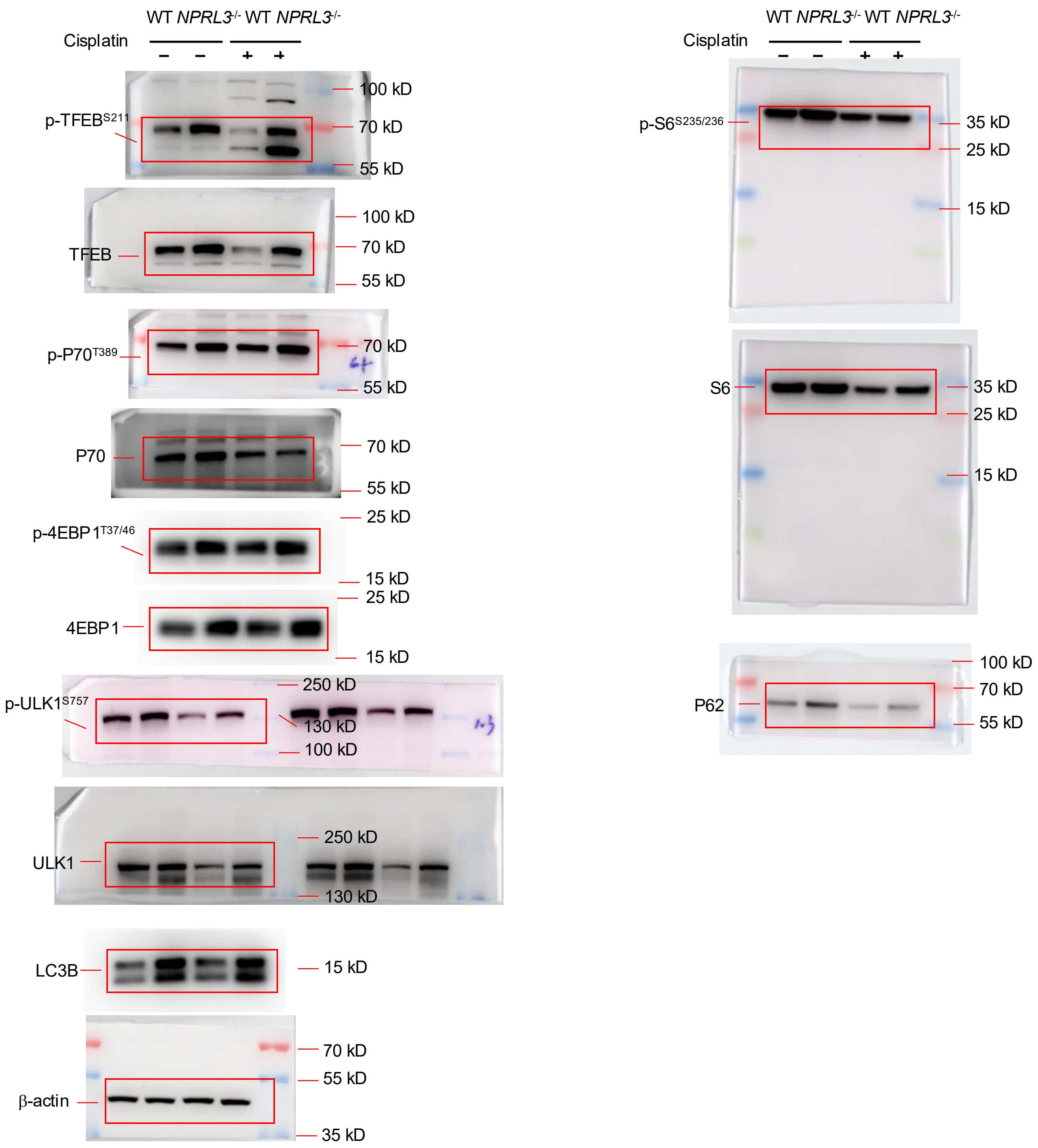

The whole uncropped images of the original blots in Fig3A.

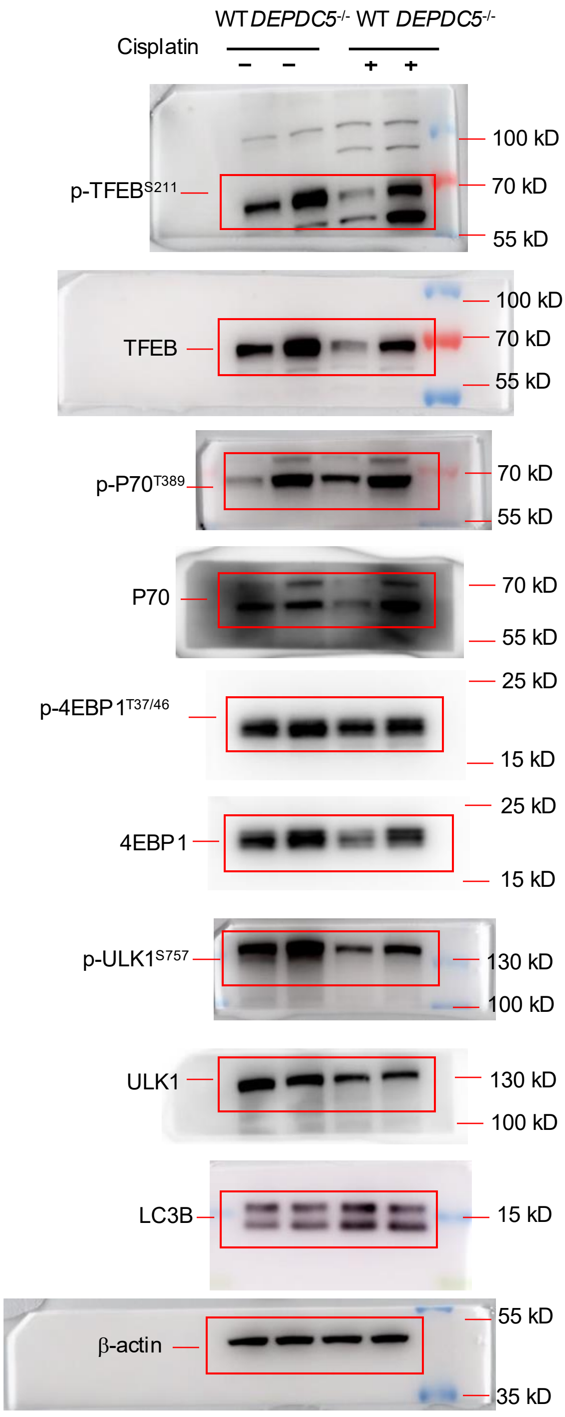

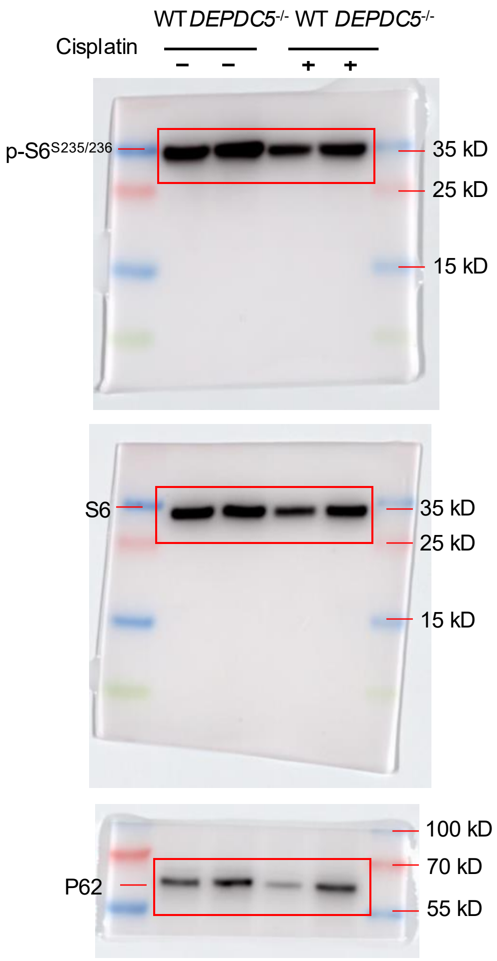

The whole uncropped images of the original blots in Fig3C.

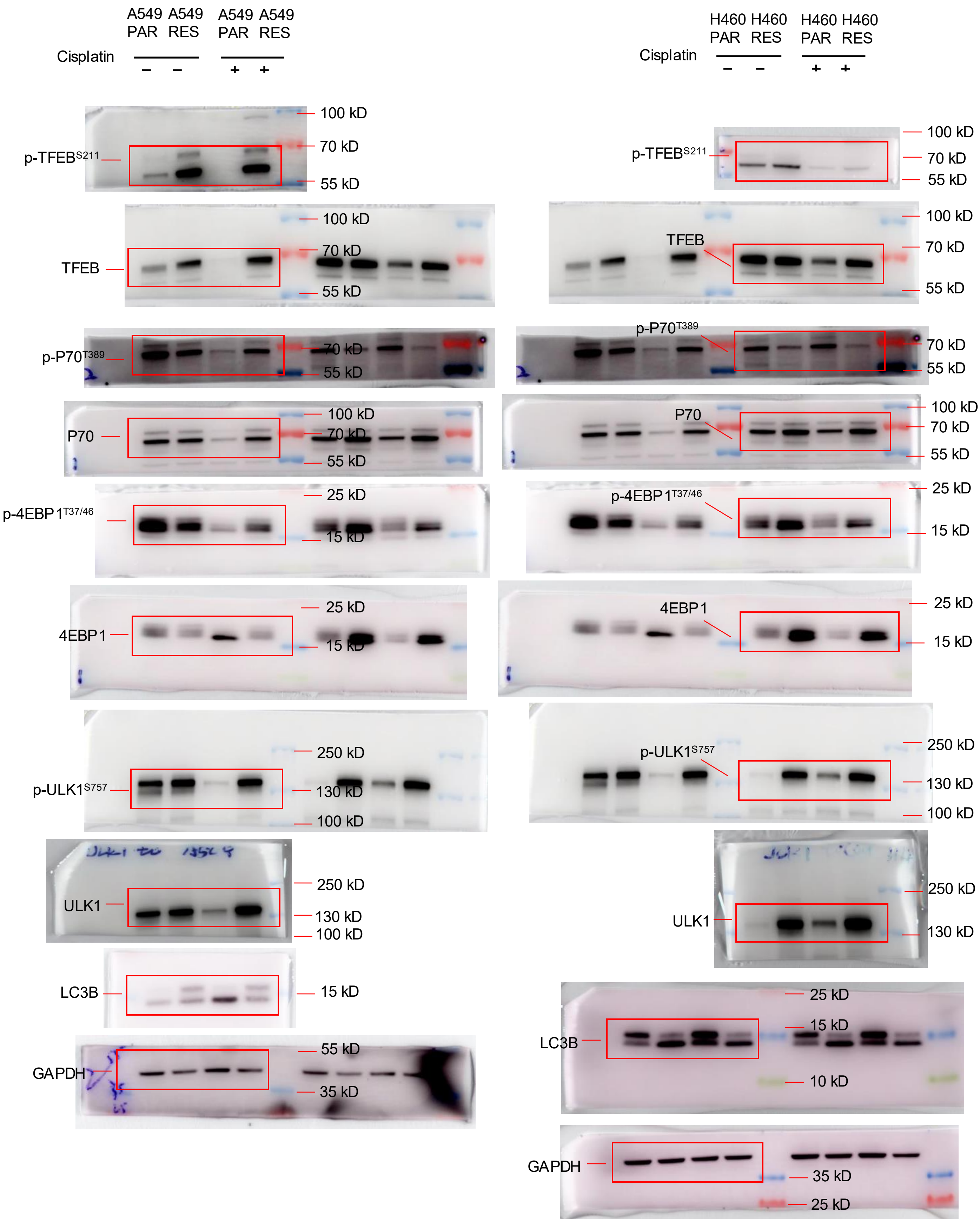

The whole uncropped images of the original blots in Fig3C.

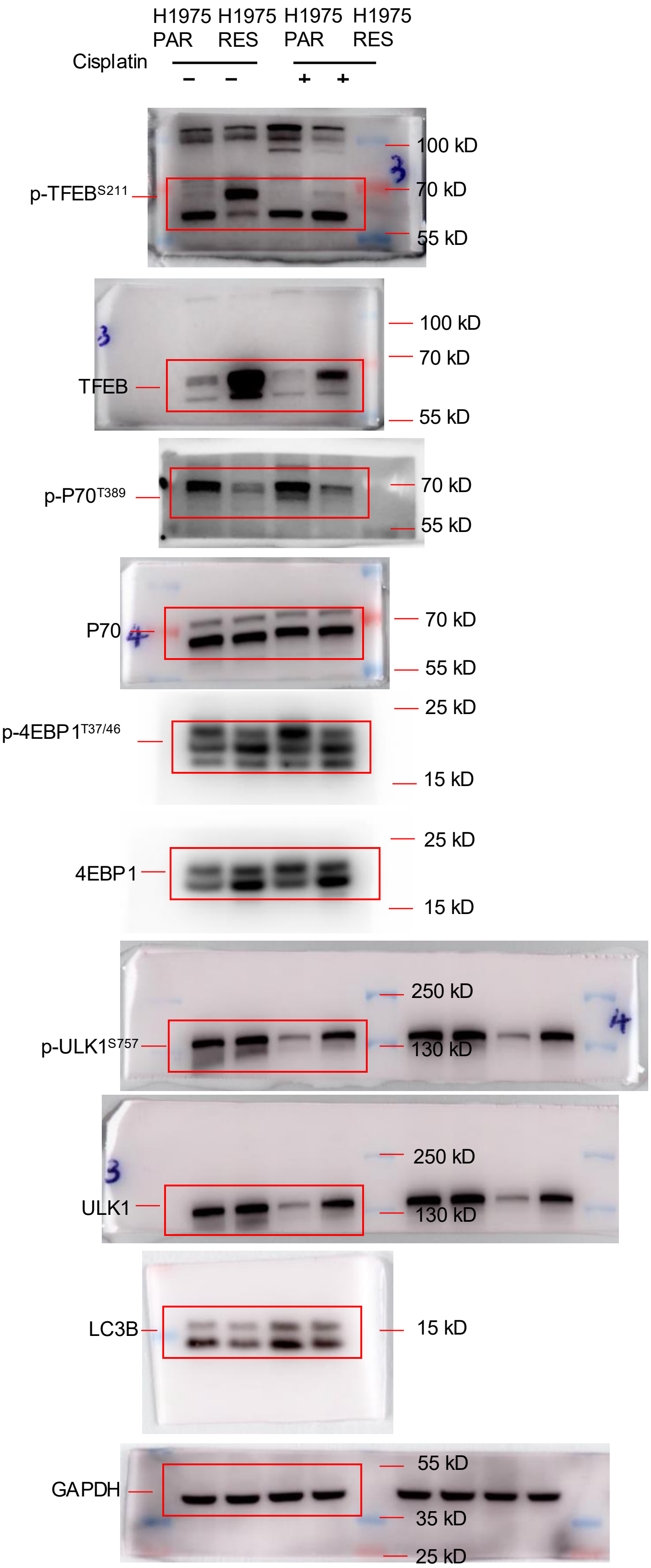

The whole uncropped images of the original blots in Fig4A and Fig4B.

Fig4A

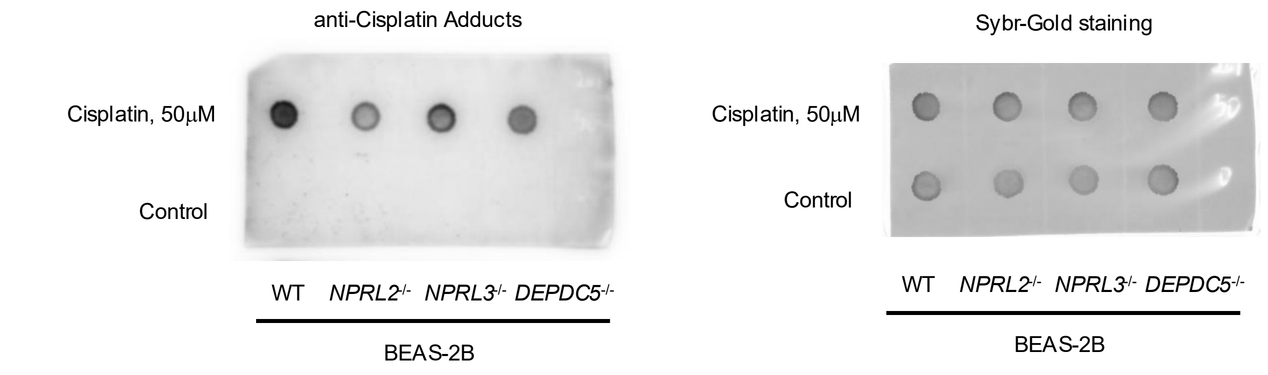

Fig4B

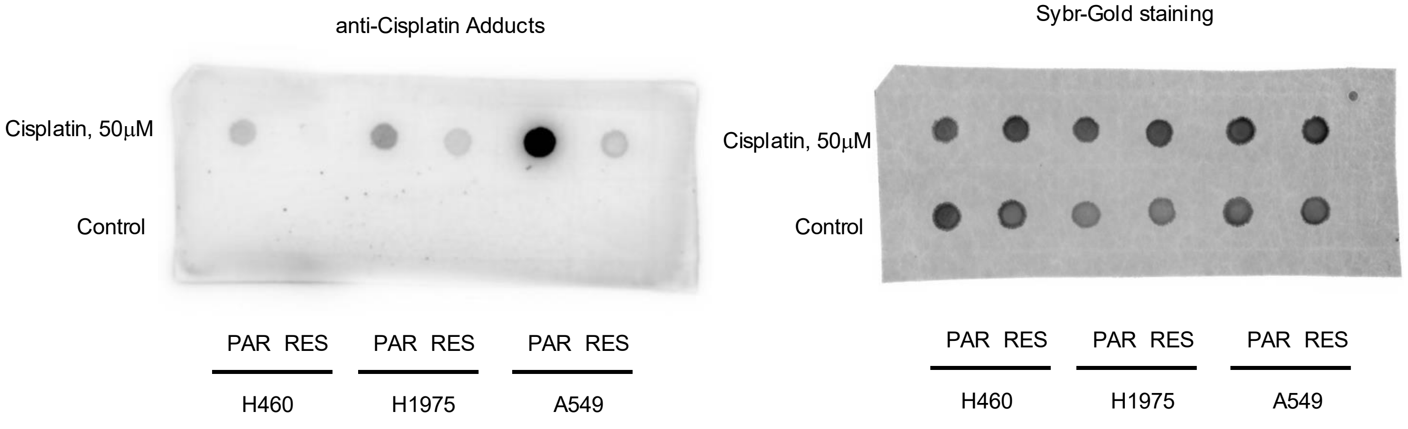

The whole uncropped images of the original blots in Fig5B.

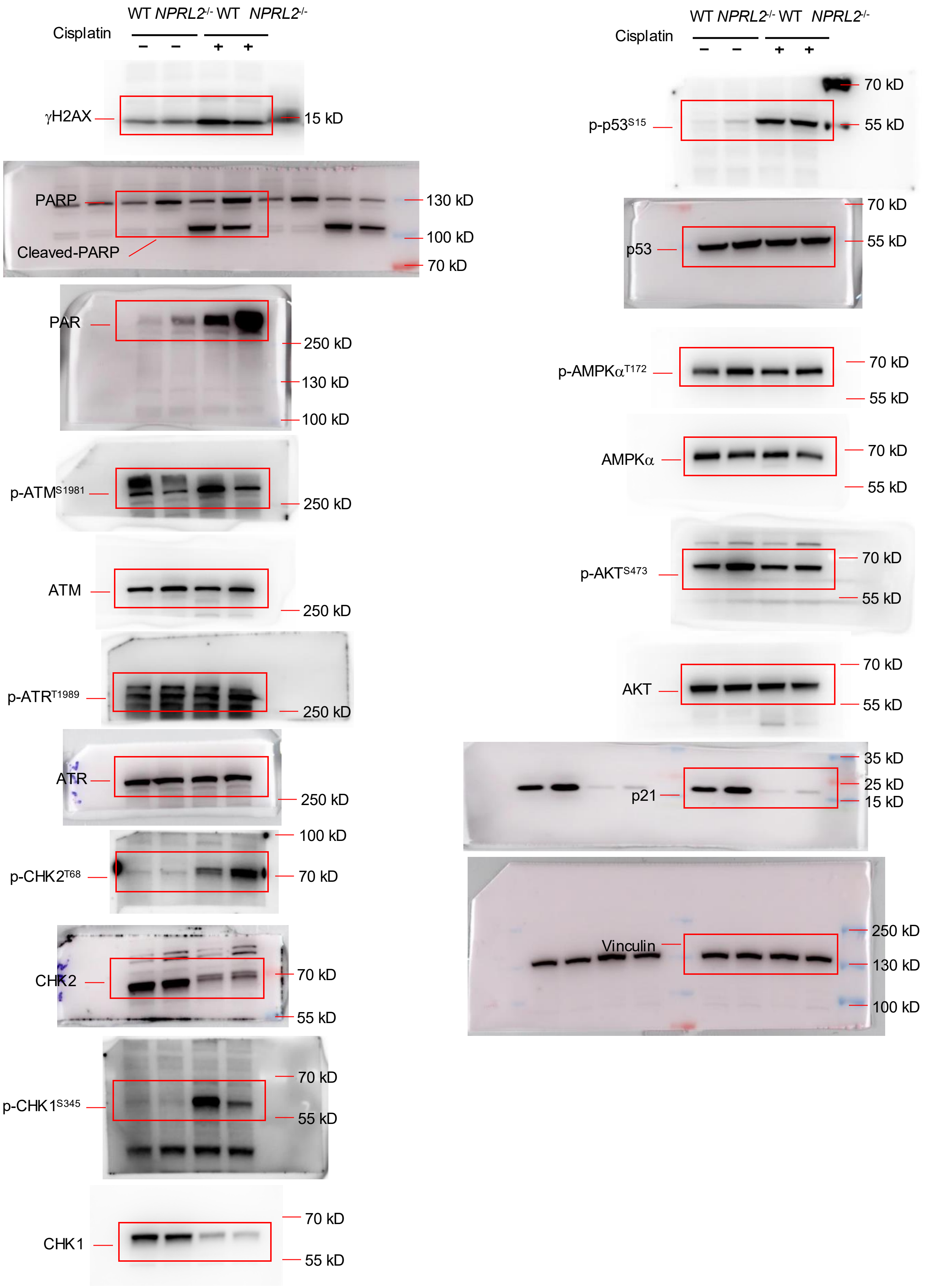

The whole uncropped images of the original blots in Fig5B.

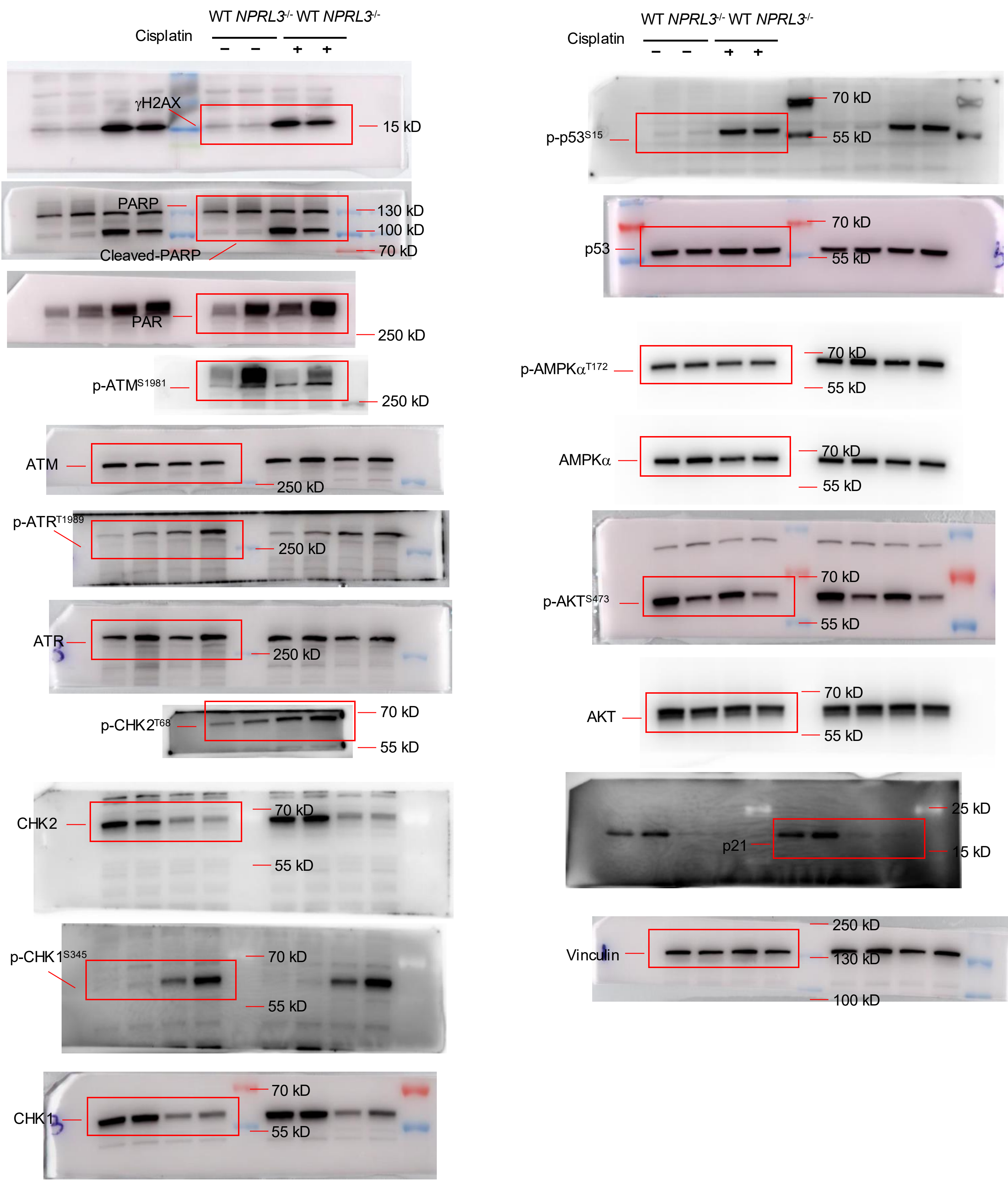

The whole uncropped images of the original blots in Fig5B.

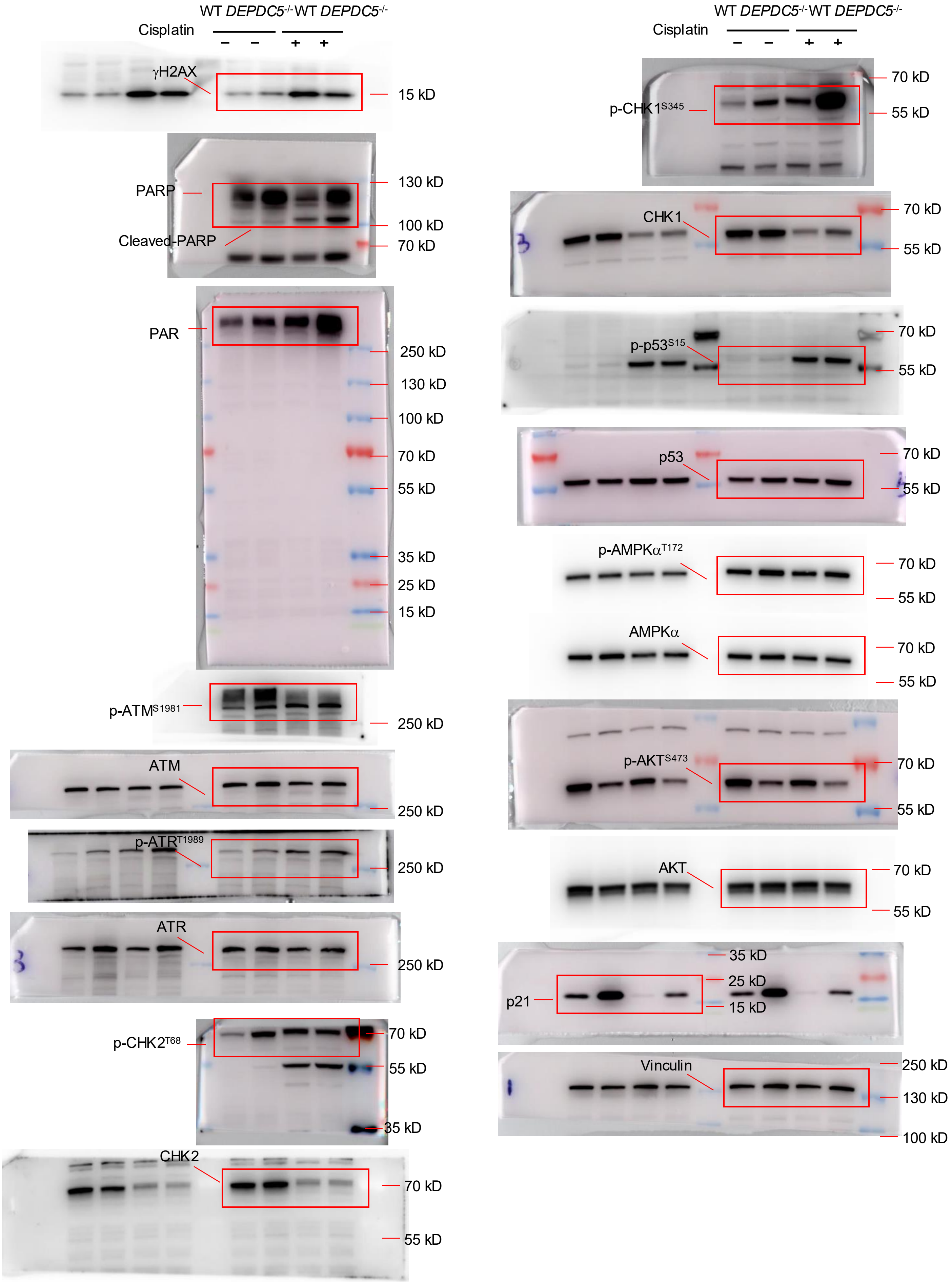

The whole uncropped images of the original blots in Fig6B.

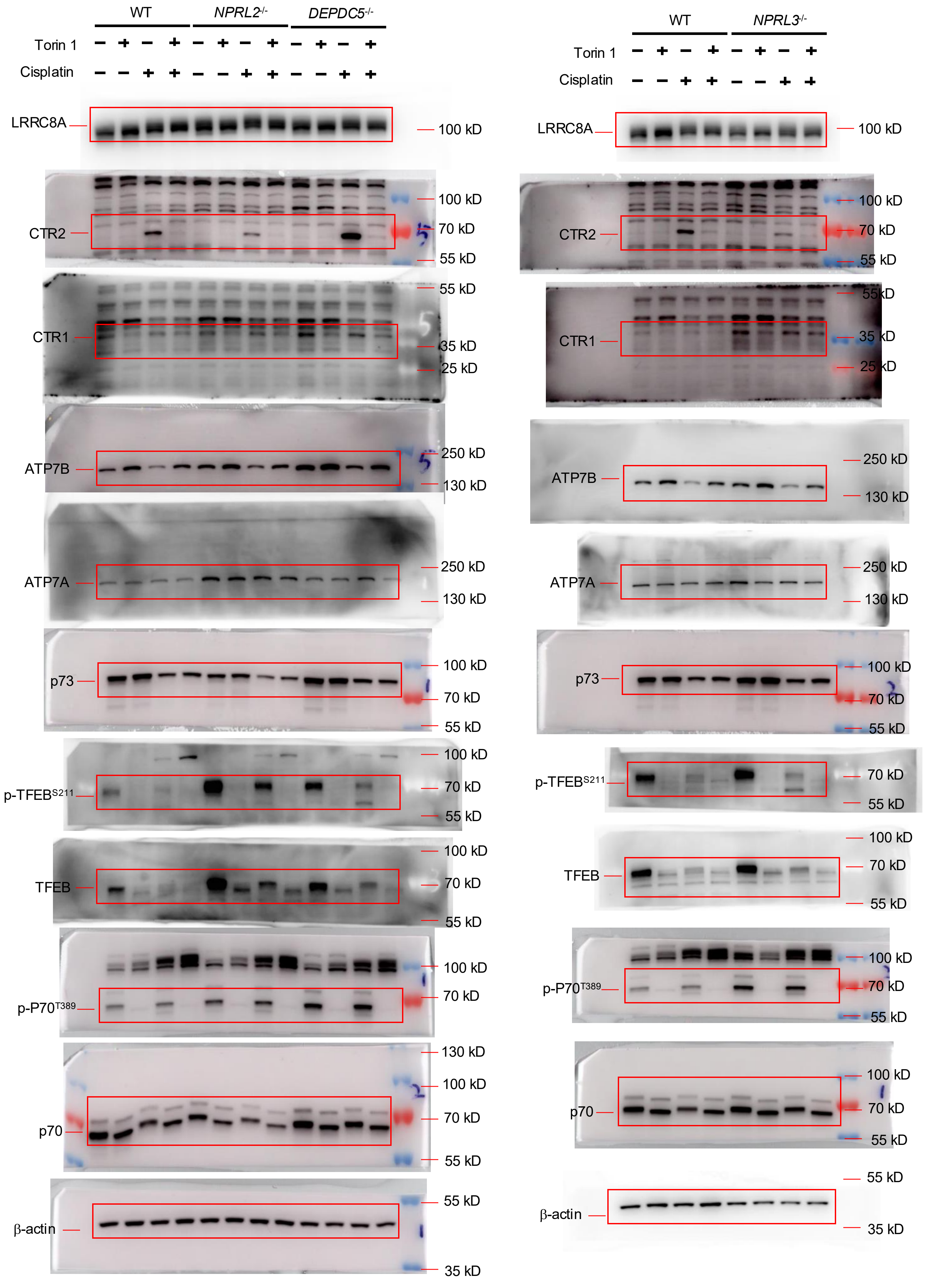

Supplement: Supplementary file 9 — Original uncropped blots [file 41419_2025_8392_MOESM9_ESM.pdf]
