# Supplementary material for: GATOR1 complex controls cisplatin sensitivity
Source: Cell Death Dis. 2025 Dec 30;17(1):58. doi: 10.1038/s41419-025-08392-4 (PMC12824275; doi:10.1038/s41419-025-08392-4)
Supplement: Supplementary file 10 — Table S1 [file 41419_2025_8392_MOESM10_ESM.docx]

**Table S1.** Comparison of differentially expressed genes and transcription factors in various GATOR1 depleted cells.

| **Cell lines** | **Total** | | | **Up** | | **Down** | |
| --- | --- | --- | --- | --- | --- | --- | --- |
|  | All genes | TFs | All genes | | TFs | All genes | TFs |
| *NPRL2^-/-^* vs WT | 841 | 66 | 131 (16%) | | 7 (11%) | 710 (84%) | 59 (89%) |
| *NPRL3^-/-^* vs WT | 1207 | 131 | 537 (44%) | | 41 (31%) | 670 (56%) | 90 (69%) |
| *DEPDC5^-/-^* vs WT | 842 | 68 | 326 (39%) | | 17 (25%) | 516 (61%) | 51 (75%) |
| *NPRL2^-/-^* cisplatin vs WT cisplatin | 3583 | 372 | 1792 (50%) | | 51 (14%) | 1791(50%) | 321(86%) |
| *NPRL3^-/-^* cisplatin vs WT cisplatin | 1556 | 140 | 980 (63%) | | 76 (54%) | 576 (37%) | 64 (46%) |
| *DEPDC5^-/-^* cisplatin vs WT cisplatin | 1601 | 101 | 656 (41%) | | 74 (73%) | 945 (59%) | 27 (27%) |
| WT cisplatin vs WT | 6648 | 561 | 3157 (47%) | | 261 (47%) | 3491(53%) | 300 (53%) |
| *NPRL2^-/-^* cisplatin vs *NPRL2^-/-^* | 6994 | 544 | 3581(51%) | | 153 (28%) | 3413 (49%) | 391 (72%) |
| *NPRL3^-/-^* cisplatin vs *NPRL3^-/-^* | 5391 | 478 | 2743 (51%) | | 272 (57%) | 2648 (49%) | 206 (43%) |
| *DEPDC5^-/-^* cisplatin vs *DEPDC5^-/-^* | 4825 | 487 | 2055 (43%) | | 306 (63%) | 2770 (57%) | 181 (37%) |
